# Peeling back the layers of coral holobiont multi-omics data

**DOI:** 10.1016/j.isci.2023.107623

**Published:** 2023-08-14

**Authors:** Amanda Williams, Timothy G. Stephens, Alexander Shumaker, Debashish Bhattacharya

**Affiliations:** 1 Microbial Biology Graduate Program, Rutgers University, New Brunswick, NJ 08901, USA; 2 Department of Biochemistry and Microbiology, Rutgers University, New Brunswick, NJ 08901, USA

**Keywords:** Natural sciences, Biological sciences, Omics

## Abstract

The integration of multiple ‘omics’ datasets is a promising avenue for answering many important and challenging questions in biology, particularly those relating to complex ecological systems. Although multi-omics was developed using data from model organisms with significant prior knowledge and resources, its application to non-model organisms, such as coral holobionts, is less clear-cut. We explore, in the emerging rice coral model *Montipora capitata*, the intersection of holobiont transcriptomic, proteomic, metabolomic, and microbiome amplicon data and investigate how well they correlate under high temperature treatment. Using a typical thermal stress regime, we show that transcriptomic and proteomic data broadly capture the stress response of the coral, whereas the metabolome and microbiome datasets show patterns that likely reflect stochastic and homeostatic processes associated with each sample. These results provide a framework for interpreting multi-omics data generated from non-model systems, particularly those with complex biotic interactions among microbial partners.

## Highlights

• Transcriptomic and proteomic data broadly capture the coral thermal stress response
• Significant discordance exists between gene expression and protein accumulation
• Metabolome and microbiome data exhibit patterns that reflect holobiont homeostasis
• Multi-omics data reflect the complex biotic interactions that sustain corals

## Graphical abstract

**Figure:**
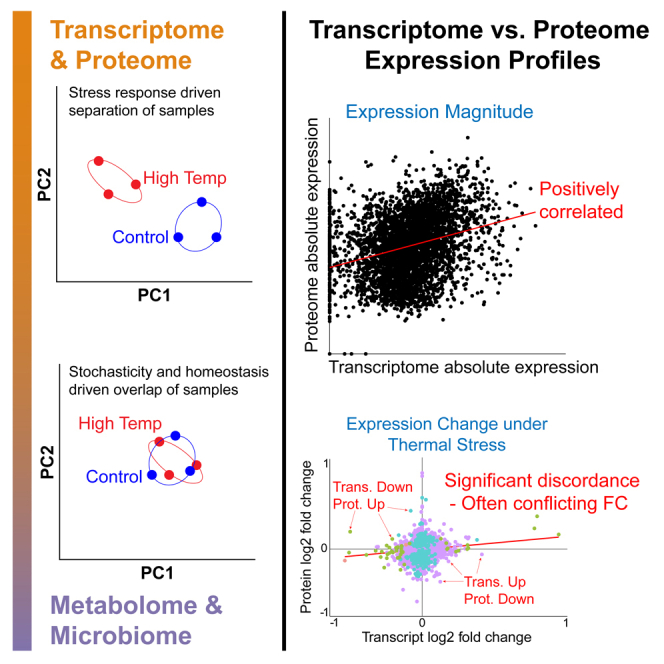

## Introduction

The devastating loss of coral reefs due to climate change has spurred ‘omics’ research to aid conservation of these valuable, biodiverse ecosystems.^1^^,^^2^^,^^3^^,^^4^ Multi-omics relies on high-throughput approaches such as genomics, transcriptomics, proteomics, and metabolomics to interrogate organismal biology. These methods were developed using data from traditional model organisms, including *Arabidopsis*, yeast, and *Escherichia coli*, which often have chromosomal-level genome assemblies and significant knowledge about gene and non-coding region functions, protein-protein interactions (PPI), and complete biochemical pathways based on genetic, bioinformatic, and biochemical data.^5^ Studies of these organisms are also well supported by omics databases and analysis tools, such as MetaboAnalyst,^6^ STRING,^7^ and KEGG.^8^ These resources allow omics data relationships to be meaningfully interpreted. Whether multi-omics can be effectively applied to non-model organisms, such as the coral holobiont, whereby individual polyps comprise a cnidarian animal host, a diverse population of large genome (1.2–2.0 Gbp in size),^9^ algal symbionts (Symbiodiniaceae), other eukaryotes such as fungi and protists, prokaryotes, and viruses, remains to be determined.

Although the cnidarian host of the coral holobiont has a simple two-tissue body plan (epidermis and gastrodermis, connected by an acellular mesoglea), reefs exist in complex, species-rich, and dynamic marine environments that make coral biology a challenging area of research. A useful approach to understand biotic interactions within this system is through metabolomics, which is rapidly developing in the coral field. However, the ratio of known to unknown metabolites in corals is still very low when compared to traditional model organisms.^10^ The same holds for genomic, transcriptomic, and proteomic data, with many of the genes and proteins identified in corals and their symbionts having unknown functions,^11^ making the interpretation of these omics data highly challenging.

In recent years, the sea anemone *Exaiptasia pallida* (also known as Aiptasia) has become a tractable model system for studying holobiont symbioses and stress responses.^12^^,^^13^^,^^14^^,^^15^ Aiptasia is globally distributed, harbors endosymbiotic Symbiodiniaceae, can be maintained indefinitely in the symbiotic or aposymbiotic (Symbiodiniaceae-free) state, and has a sequenced genome.^12^ Because Aiptasia can be propagated sexually and asexually in laboratory tanks, large clonal populations are available for use in high-replicate time-course experiments and genetic studies.^16^ These characteristics would potentially ameliorate many of the current obstacles in coral omics data analysis, such as the functional characterization of ‘dark’ (i.e., of unknown function) genes and metabolites, developing metabolic maps specific to cnidarians, and elucidating PPIs. Yet, regardless of the potential of Aiptasia as a Cnidarian model system, this species currently does not have the same resources, background information, or data analysis tools available for multi-omics data analysis and integration as do traditional model organisms. Furthermore, insights from Aiptasia biology cannot always be applied to corals due to the absence of biomineralization in the former, the relatively shorter lifespan (coral colonies can persist for hundreds of years^17^), and a smaller genome size.^12^ Thus, understanding the capacity and limitations of omics techniques applied to the coral holobiont, as well as the cases in which Aiptasia may or may not serve to improve omics data interpretation, will aid the progress and utility of coral multi-omics research.

Here, novel proteomic and prokaryote microbiome 16S-rRNA amplicon data were analyzed, along with existing transcriptomic and metabolomic data from the stress-resistant Hawaiian coral *Montipora capitata*.^18^ Whereas 16S-rRNA community profiling (e.g., in contrast to prokaryotic metagenomic data) is limited in terms of the questions it can address about changes in the functional ecology of a community, the information gained by this type of analysis provides a useful tool that, in combination with other omics data, can be used to generate hypotheses for follow-up studies. Profiling of the 16S-rRNA community (which we consider here to be an omics approach) is also widely used to study the bacterial component of the coral holobiont, therefore understanding how these data can be effectively integrated with other approaches is of high interest. Given these existing data, our goal was to ascertain how well different layers of multi-omics data can be integrated in *M. capitata* samples derived from a single experiment. Using the available genome assembly for this coral species as a foundation,^19^ multiple animal genotypes were subjected to a 5-week thermal stress regime. Control and treatment samples were collected at three time points, which coincide with initial thermal stress, the onset of bleaching, and four days after initial bleaching (Figure S1).^18^

We find that transcriptomic and proteomic data broadly capture the thermal stress response of *M. capitata*, albeit the specific genes identified are often not shared across datasets. We also find that whereas the overall magnitude of expression of these datasets is positively correlated, there is significant discordance vis-à-vis the extent of differential change when comparing control and treatment conditions, which is lessened under stress. In contrast, the metabolite and microbiome data show patterns that likely reflect the complex nature of the holobiont, with these data impacted by homeostatic processes and by fine-scale interactions between the holobiont and its proximate environment. These results provide insights into the different behaviors of multi-omics data and their interpretation when studying complex ecological systems such as corals.

## Results

### Proteome and transcriptome data

There were 4036 *M capitata* proteins (3882 [96.18%] high confidence identifications) which had peptides identified in at least one of the proteomic samples (https://zenodo.org/record/6861688 and Table S1). Of these proteins, 2760 (68.38%) had KO numbers assigned, with 414 (15%) of these belonging to at least one of the major biochemical pathways presented in Table S2. In comparison, of the 63,227 predicted proteins in the *M. capitata* genome, 18,684 (29.55%) have annotated KO numbers and 1925 (10.3%) belonged to at least one major biochemical pathway.

**Figure 1:**
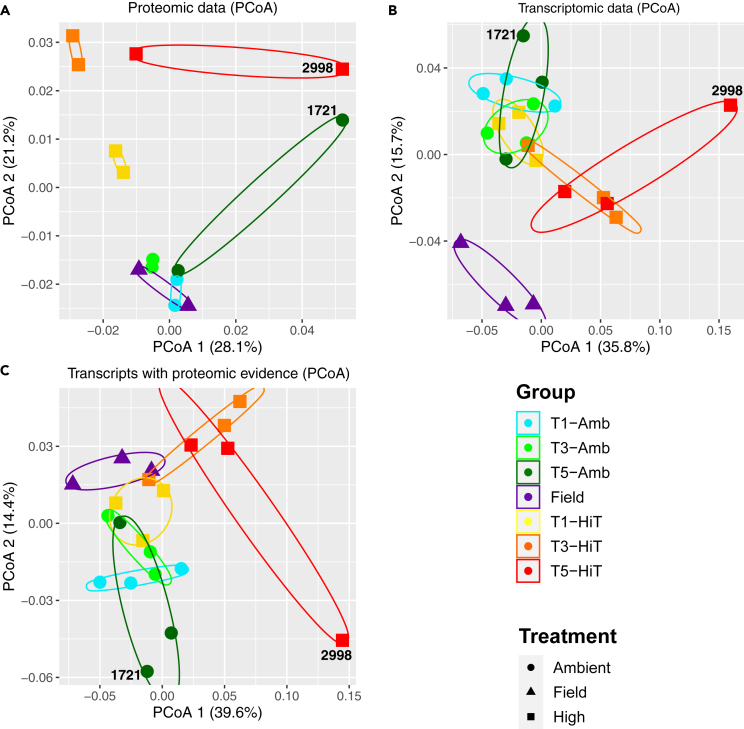
Relationship between proteomic and transcriptomic samples from one genotype (MC-289) of *M. capitata* (A–C) PCoA plots generated using the (A) proteomic data, (B) transcriptomic data, and (C) transcripts with proteomic evidence, respectively. PCoA plots are based on Bray-Curtis distances between all samples in the corresponding dataset. The shape of each point corresponds to the treatment (ambient, high temperature, or field samples) and the color corresponds to the treatment and time point at which each sample was collected; a legend with this information is shown in the bottom right corner of the image. Samples from the same condition are grouped with colored ellipses. The amount of variance explained by each axis in each plot is displayed in parentheses. Samples derived from mislabeled genotypes are annotated with their respective plug IDs (2998 for MC-289_T5-HiT_2998 and 1721 for MC-289_T5-Amb_1721).

The PCoA plots of the proteomic and transcriptomic data (Figures 1A and 1B, respectively) from a single *M. capitata* genotype (MC-289) demonstrate that samples group well by time point and treatment. The field samples tend to group closely with the ambient samples, because this was not a period of bleaching in Kāne’ohe Bay, O’ahu where the experiments were carried out. This result is supported by sPLS-DA (Figures S2A and S2B) and PCA (Figures S2D and S2E) plots, which also show the samples in each dataset grouping by time point and treatment. Generally, samples from the same group are positioned close together across the different analysis plots, particularly in the proteomic dataset, although, there are some outliers (i.e., MC-289_T5-HiT_2998 and MC-289_T5-Amb_1721 in Figures 1A and 1B). Genotyping showed that all but two (MC-289_T5-HiT_2998 and MC-289_T5-Amb_1721) of the transcriptome samples have high (∼98%) proportions of shared SNPs, suggesting that they are all from the same genotype (as expected). The two samples share <90% of their SNPs with each other and with the other samples, suggesting that they are from different genotypes (Figure S3). This is potentially due to a mix-up in sample labeling. Inclusion of these samples in downstream analysis is unlikely to greatly affect our interpretations due to the statistical approaches used that take sample variance into account, and because we focus our discussion on the samples from TP1 and TP3, which are all confirmed to be from the same genotype.

When these ordination methods (PCoA, PLS-DA, and PCA) were applied to only transcripts from genes with proteomic evidence (Figures 1C and S2C–S2F, respectively), they all showed that the samples group well by time point and treatment, and are roughly congruent with the relative positioning of the samples in the full proteomic and transcriptomic datasets (although the plots are more similar to the latter than the former). For example, in the proteome ordination plots (Figures 1A, S2A, and S2D), there is relatively little variation (excluding the mislabeled samples) along each axis between the replicates from each group compared to the variation between groups. In contrast, the transcriptome, and transcripts with proteomic evidence, ordination plots show greater relative variation between samples from the same group and lower variation between groups, with sample from the same condition (e.g., ambient) often overlapping on both axis (Figures 1B, 1C, and S2B–S2F). This suggests that whereas the same general stress response pattern is present in both datasets, evidenced by the same relative relationship between the replicates and groups, there are numerous differences. These results provide evidence that the dynamics of the stress response in each omics data layer differ, even for the same set of genes. The PERMANOVA results (Table S3) show that none of the factors assessed contribute significantly to the variance between groups, however, time point does tend to have the lowest significance across the datasets, which is congruent with the strong association between the field, ambient (all time points), and TP1 high temperature treated samples, and the more distant association between the TP3 and TP5 high temperature treated samples in the ordination results (Figure 1).

### Protein abundance-transcript expression level correlation

**Figure 2:**
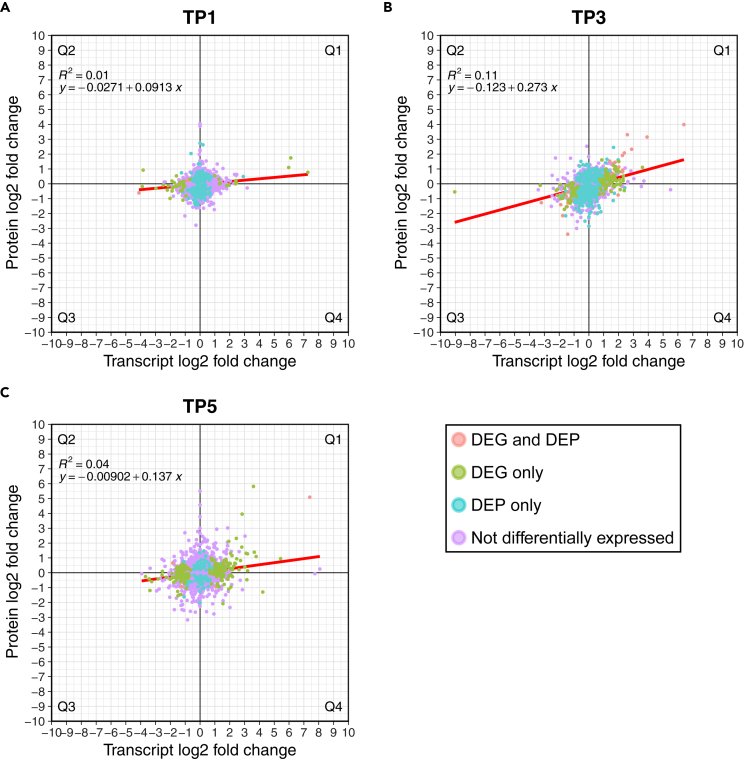
Differences in protein and transcript expression FC for each gene identified in the MC-289 proteomic data (n = 4036) (A–C) Fold change of transcripts (x axis) and protein (y axis) expression values between high vs. ambient temperature samples for time points (A) TP1, (B) TP3, and (C) TP5. Each plot is divided into four quadrants (Q1-Q4), Q1 contains genes with positive protein and transcript FC values, Q2 contains genes with positive protein and negative transcript FC values, Q3 contains genes with negative proteins and transcript FC values, and Q4 contains genes with negative protein and positive transcript FC values. The fold change values for all genes across the three time points are presented in Table S1. A trend line (red) is fitted through the data with associated R^2^ value and line formula shown in the top left corner.

For genes with proteomic evidence, the log_2_ fold change (FC) abundance differences between the protein and associated transcript, between the ambient and high temperature treated samples at each time point, were quantified (Figure 2). Each plot in Figure 2 is divided into four quadrants (Q1-Q4), with genes in Q1 having positive protein and transcript FC values, Q2 having positive protein and negative transcript FC values, Q3 having negative protein and transcript FC values, and Q4 having negative protein and positive transcript FC values. A trend line fitted to the data from each time point shows that at TP1 there is little correlation (R^2^ = 0.01) between the gene, transcriptome, and proteome FC values. This correlation increases at TP3 (R^2^ = 0.11) and to a lesser extent TP5 (R^2^ = 0.04), with there being more variation in the magnitude of FC values at TP3 and TP5 compared to TP1. In addition, whereas the genes at TP1 form a roughly circular cloud centered around zero transcript and protein FC, there is a more pronounced spread at TP3, and to a lesser degree TP5, of genes through Q1 and Q3 (i.e., the quadrants where transcript and protein FC directionality correlate). Normalized protein and transcript expression levels of proteins identified in the proteomic data were plotted for each combination of time point and treatment (Figure S4). There was a positive, but weak correlation (R^2^ = 0.07–0.11) between the normalized protein and transcript expression levels of proteins identified in the proteomic data across all samples, regardless of treatment or time point.

A list of 138 genes associated with thermal stress in corals was compiled and used to further assess the correlation between transcript and protein expression (Table S1).^16^^,^^20^^,^^21^^,^^22^^,^^23^ Whereas this gene list is enriched for thermal stress-response genes, many general stress-response genes are also included in the target set. Only 55 of the stress-response genes have significant changes in either transcript or protein expression between the ambient and high temperature treated samples at any of the time points. As expected, TP3 and TP5 had more stress-response genes that are differentially expressed in the transcriptome (TP1 = 5/138; TP3 = 20/138; TP5 = 16/138), because these time points showed a change in the color score of the coral nubbins (Figure S1). That is, the high temperature samples showed signs of thermal stress at these time points. TP3 also showed more stress-response genes that are differentially expressed in the proteome (TP1 = 8/138; TP3 = 29/138; TP5 = 6/138). There are only nine stress-response genes that are both a DEG and DEP, all of them are at TP3, and all of them have the same FC directionality in the transcriptome and proteome. Further, in the total proteome dataset there were 50 genes that are DEPs and DEGs at TP3, and only three didn’t share the same FC directionality. We chose to focus on the expression profile of genes at TP3 because it was the time point at which the corals showed signs of bleaching both in terms of their physiological and transcriptomic responses,^20^ and TP1 because it showed little signs of bleaching and represents are more stable homeostatic state. Additionally, the samples from TP1 and TP3 were confirmed to be from the same genotype, which is not the case for TP5, which likely explains the lack of DEPs at this time point. At TP1, the majority of stress-response genes (80/138 [58%]) had transcript and protein FC values that were in the same direction (i.e., both positive, or both negative). At TP3, even more of the stress-response genes (92/138 [66.7%]) had transcript and protein FC values that were in the same direction. In the total proteome dataset, at TP1 2013/4036 (49.88%) and at TP3 2250/4036 (55.75%) genes had FC values that were in the same direction.

### Polar metabolomic data

**Figure 3:**
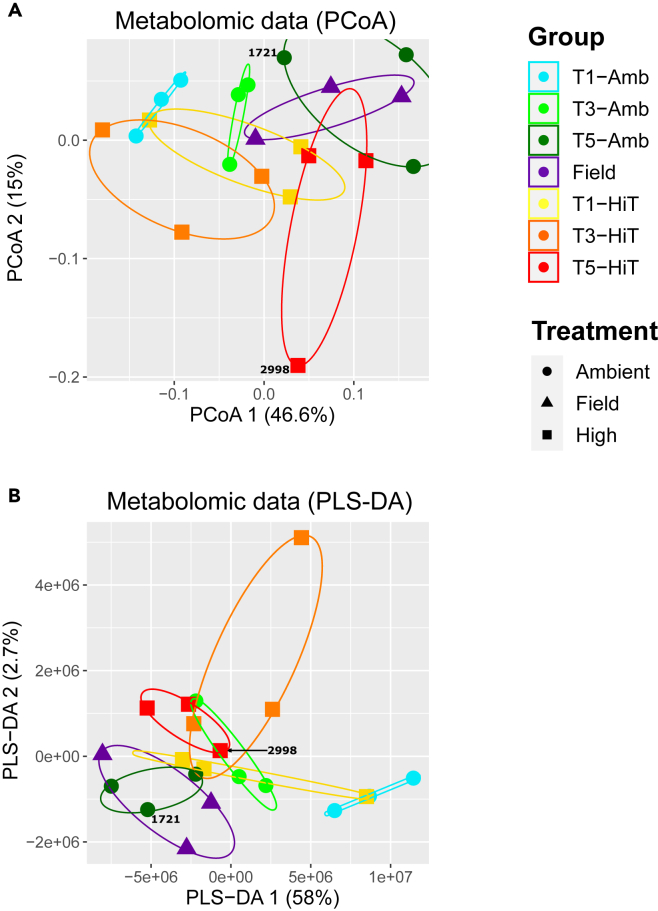
Relationship between metabolomic samples from one genotype (MC-289) of *M. capitata* Data presented as (A) PCoA and (B) PLS-DA plots. The PCoA plot is based on the Bray-Curtis distances between all samples in the dataset. The shape of each point corresponds to the treatment (i.e., ambient, high temperature, or field) and the color corresponds to the treatment and time point at which each sample was collected; a legend with this information is shown on right of the image. Samples from the same condition are grouped with colored ellipses. The amount of variance explained by each axis in each plot is displayed in parentheses. Samples derived from mislabeled genotypes are annotated with their respective plug IDs (2998 for MC-289_T5-HiT_2998 and 1721 for MC-289_T5-Amb_1721).

The polar metabolomic dataset generated using positive ionization contained 11,649 peak features after normalization and filtering. Both supervised (sPLS-DS; Figure 3B) and unsupervised (PCoA and PCA; Figures 3A and S5, respectively) methods, when applied to the filtered metabolite features for a single *M. capitata* genotype (MC-289) show that samples group by time point and treatment, but without the clear separation (driven by a given factor) between groups observed in the proteomic and transcriptomic ordination plots (i.e., the groups largely overlap; Figures 1 and S2). Combining the metabolomic data from all four genotypes into a single analysis does not change this result (Figure S6), with no separation observed between samples from the different genotypes. The PERMANOVA results show that time point and treatment contributed significantly (p value <0.05) to the variation in the metabolomic data. Genotype is a significant factor when data from all four *M. capitata* genotypes are analyzed, however, none of the interaction terms involving genotype or time point were significant (Table S3).

### Microbiome 16S rRNA data

**Figure 4:**
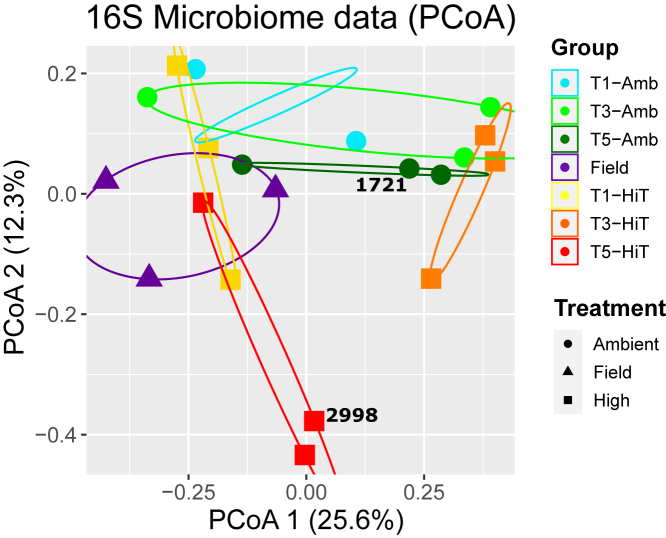
Relationship between 16S microbiome samples from one genotype (MC-289) of *M.capitata presented as a PCoA plot based on Bray-Curtis distances* The color of each point corresponds to the treatment and time point at which each sample was collected; a legend with this information is shown on right of the image. Samples from the same condition are grouped with colored ellipses. The amount of variance explained by each axis in each plot is displayed in parentheses. Samples derived from mislabeled genotypes are annotated with their respective plug IDs (2998 for MC-289_T5-HiT_2998 and 1721 for MC-289_T5-Amb_1721).

A total of 12,432 ASVs were produced from microbiome 16S rRNA sequencing data (https://zenodo.org/record/6861688). The Kruskal-Wallis rank sum tests and pairwise comparisons using Wilcoxon rank sum tests revealed only time point as having a significant impact on α-diversity metrics (p values = 0.00242 and 0.005086 for Shannon and Simpson metrics, respectively). Similarly, when investigating β-diversity, time point was the only significant factor found in ANOSIM tests and the most significant factor in PERMANOVA tests conducted on Bray-Curtis distance matrices (p value = 0.01 and 0.009, respectively; Table S4). PSL-DA, PCA, and PCoA further demonstrates that bacterial community compositions of the samples are quite dissimilar, even among replicate samples from the same treatment, time point, and genotype (Figures 4, S7, and S8). In addition, we find an increase in the α-diversity of the samples from each of the four genotypes over time until TP3 (Figure S9). This trend is also reflected in the pairwise Wilcoxon tests between the Field vs. TP3 and TP1 vs. TP3 time points, which have significant (p value <0.05) associations for the Observed and Simpson’s metrics. This increase in diversity metrics between TP3 and earlier time points can also be observed in the abundances of bacterial phyla across the samples from the four genotypes (Figure S10; Table S5).

### Correlation between the microbiome and metabolome datasets

At the phylum, class, and order levels, the only significant correlation (*r* = 0.70785488; p value = 5.503 x 10^−8^, 1.276 x 10^−7^, 3.389 x 10^−7^, respectively) that was found is between *Marinimicrobia* (SAR406 clade) and compound 10951 (*m/z* = 456.11676, *rt* = 1.613358; Table 1). At the family level significant correlations were found again between *Marinimicrobia* and compound 10951 (*r* = 0.70785488, p value = 5.82 x 10^−7^). Weaker but significant correlations were also found between compound 9802 (*m/z* = 200.961411, *rt* = 5.271) and family *LWQ8* (Patescibacteria phylum; *r* = 0.567811104, p value = 0.044185845), as well as between compound 11436 (*m/z* = 596.334717, *rt* = 6.351) and family *Coleofasciculaceae* (cyanobacteria; *r* = 0.565262299, p value = 0.044185845).

**Table 1:** Spearman correlation coefficients for those bacterial families for which significant correlations with metabolites were found

Family	groupID 9802	groupID 10951	groupID 11436
Coleofasciculaceae	−0.074	−0.084	0.565 (**0.044**)
LWQ8	0.568 (**0.044**)	0.137	−0.076
Marinimicrobia (SAR406 clade)	0.008	0.708 (**5.82 x 10**^**−**^**^7^**)	0.193

Adjusted p values for significant correlations are provided in bold within parenthesis after the correlation coefficient.

## Discussion

### Discordance between coral animal transcript and protein abundance

Analyses conducted using a single *M. capitata* genotype shows that the expression patterns of validated coral animal proteins and transcripts are strongly influenced by the time point and treatment at which the samples were collected (Figures 1 and S2). Whereas there are no factors that have significant effects on the proteomic and transcriptomic datasets (Table S3), time point did have the lowest significance values compared to treatment (also observed with the 16S microbiome and metabolome data). This is unsurprising given the strong association between the field, ambient (all time points), and TP1 high temperature treated samples in the ordination plots (Figures 1 and S2). There is relatively little variation between samples from the same treatment group in the proteomic data compared to the transcriptomic data. This increased variation in gene expression at the transcript level could result from environmental and temporal (i.e., treatment) factors acting on the large number of genes detected in this dataset, as well as the dynamic feedback between transcripts and proteins (discussed in more detail in a later section) and inherent “noise” in the expression of genes in the genome.^24^^,^^25^ In contrast, environmental factors have a less significant impact on the proteomic data. It is noteworthy that many of the proteins detected in this dataset are critical for cellular function (supported by their higher rate of KO number assignment) and large changes in their abundance would likely be harmful or potentially fatal to the organism. This suggests that transcriptome data capture the corals immediate response to stress, whereas proteome data capture the longer-term response of the animal and are less impacted by gene expression variation.

Interestingly, when the same approaches (i.e., PCoA, PLS-DA, and PCA) are applied to the data from transcripts with proteomic evidence, the relative positioning of the different sample groups, and the level of variation between samples within each group, are highly similar to the full transcriptomic dataset (Figures 1 and S2), though some clear differences are apparent (such as the positioning of the T1-Amb and Field samples in Figure S2C). This suggests that while the proteomic and transcriptomic datasets are both informative about the coral thermal stress response, they are differently impacted by external factors and have different expression dynamics that lead to a disconnect between the observed stress response of the same gene in both datasets (Figure 2; Table S1). Furthermore, despite the fact that transcripts and proteins from the same gene exhibit a weak but positive correlation, in terms of the magnitude of their accumulation (Figure S4), which is consistent with previous observations,^26^ the FC of their normalized expression levels between ambient and high temperature conditions for each time point differ significantly (Figure 2). The change in distribution of genes along both axes over the time points, specifically the spread of genes in Q1 and Q3 at TP3 compared to TP1, suggests that the link between protein and transcript FC may be stronger under stress, although there are still a significant number of genes with conflicting FC values (i.e., those in Q2 and Q4). That is, when the organism is not under thermal stress (i.e., TP1) the system is at homeostasis in both the ambient and high temperature samples, with stable rates of protein and transcript degradation and synthesis. Under these conditions the effects of micro-environmental (i.e., specific to each sample) and expression noise will have greater effects, leading to the weaker correlation between the FC of the two datasets. When the organism is under stress, protein degradation (observed in Aiptasia under thermal stress^27^) and differential regulation of stress-related genes moves the system out of homeostasis, increasing the differences between the ambient and high temperature treatment groups. The increased differential expression of proteins under these conditions has a corresponding effect in the transcriptome (e.g., transcript expression increases to accommodate increased protein syntheses, which is a result of increased protein degradation), which results in the increased correlation between the FC of the two datasets. This is apparent, given the differences between TP1 and TP3, specifically the number of DEPs and DEGs, the FC magnitudes, and the distribution of points in Q1 and Q3. Furthermore, the increase in the number of genes with FC values with shared directionality (i.e., both positive or both negative in the transcriptomic and proteomic datasets) does increase at TP3 (from 52.75% to 59.3%) across all genes with proteomic evidence, with this effect even more pronounced in just the selected stress-response genes (from 58% to 67.4%).

These results demonstrate that, in *M. capitata*, protein presence and abundance do not necessarily correlate to transcript expression, even for genes shown to be related to thermal stress (Table S1). However, this effect may be highly dependent on treatment conditions, with stress likely to result in stronger correlations. There are many well described processes that can lead to discordance between the proteome and transcriptome. For example, the shorter half-life of mRNA when compared to the encoded protein, particularly if the mRNA is modified or translationally enhanced (this might explain the genes in Q2). Post-transcriptional regulation (RNA silencing via miRNA, increased transcript turnover, or transcriptional regulators), increased protein turnover (protein degradation, either deliberate or caused by misfolding), and protein buffering could also explain this discordance (and could explain the genes in Q4).^26^^,^^28^ This discordance is not surprising and is well characterized in model organisms.^29^^,^^30^^,^^31^^,^^32^ Our results therefore demonstrate the utility of these omics datasets but underline why both transcript and protein abundance data are needed to gain a more meaningful understanding of coral biology.

Our experimental design does not allow for the exploration of the lag between changes in the expression of a transcript and the corresponding change in protein abundance because the timescale of this study was days to weeks, which is typical of coral stress experiments. To explore this issue, transcript and protein abundance samples would need to be collected multiple times per hour. Regardless, our results demonstrate that at any given time, transcript abundance cannot be assumed to serve as an accurate proxy for protein abundance. Gene expression patterns can, of course, be used as biomarkers if they show a strong correlation with stress, however, proteomics or protein-specific assays are required to ascertain the true abundance of proteins. These omics data layers have well developed and extensive tools and resources available, further enhancing their usefulness when applied to non-model systems.

### Multiple experimental factors affect polar metabolite levels

Although the polar metabolomic samples did group by time point and treatment in the supervised (PLS-DA) and unsupervised (PCoA and PCA) ordination plots, the groups often overlap in the single genotype (MC-289) dataset (Figures 3 and S5), and even more so when combining samples from all four genotypes (Figure S6). These results suggest that total metabolomic data, which includes compounds produced by all members of the coral holobiont, rather than only reflecting the effects of the major factors (i.e., time point and treatment) on the coral host, is significantly influenced by multiple aspects of the holobiont environment, including the complex interactions between the holobiont, host genotype, and the external environment, as well as stochastic and homeostatic processes acting on the holobiont. For example, metabolites such as nucleic acids, organic acids, and organooxygen compounds change very little when corals are exposed to thermal stress.^33^ This may be an outcome of metabolic homeostasis in the holobiont, driving the regulation of the levels of these important compounds. In other words, significant changes in the abundance of these metabolites are likely to be deleterious (or even fatal) to the coral, and even under severe stress, their levels may not change significantly. In contrast, previous studies of metabolite data have shown that the accumulation of specific dipeptides (and other metabolites) is significantly correlated with increasing exposure to thermal stress in *M. capitata* and *Pocillopora acuta*, regardless of animal genotype.^18^ The role of amino acids in stress signaling is well known in animal systems.^34^

Our study focused on small polar molecules which change rapidly in response to metabolic activity and exchange between the organism and its environment.^35^ However, other extraction and analysis protocols, which target primary metabolites, such as lipids and fatty acids, can also provide complementary information about the health of the holobiont (particularly given that the algal symbionts may use lipids to transfer energy from photosynthesis to the host^36^). It should be noted that coral metabolomic data are challenging to interpret for a number of reasons: 1) the presence of many “dark” metabolites limits the utility of untargeted data^18^^,^^37^ (i.e., only a few hundred coral metabolites out of tens of thousands, if not hundreds of thousands, that are detected can be identified using available databases^10^); 2) the widely differing metabolite turnover rates necessitates a large number of sample replicates from the same colony to gain statistical significance; and 3) the inability to determine which holobiont component produces each metabolite. Aiptasia may offer an important avenue for addressing some of these problems because it can be maintained in aposymbiotic and symbiotic forms, allowing for the holobiont manipulation required to explore the production and use of specific metabolites.

### What are the factors that drive shifts in the microbiome profile?

The holobiont microbiome amplicon data show little association with treatment (Figures 4, S7, and S8), but do show a significant (p value <0.05) change in α- and β-diversity metrics over the course of the study (Table S4). This result is likely not explained by a prokaryotic composition that reflects vastly different habitats of origin because these *M. capitata* colonies were collected from the same reef and the difference in the composition of the genotypes was not statistically significant (Table S4). Therefore, it is likely that change in the microbiome composition of samples over the experiment reflects multiple factors, including, gradual acclimation of the samples to the indoor tank environment that used water drawn from Kāneohe Bay. In addition, the constant turnover (shedding) of the coral mucus layer every few hours and selection for holobiont fitness may have homogenized the microbiome community in different colonies. Despite significant variability in the composition of the prokaryotic microbiome between different coral species, between species across a broad geographic range, and even across a single coral colony,^38^ these prokaryotes likely play an important role in coral biology and the holobiont response to stress.^39^^,^^40^

### The diversity of microbial species makes integration challenging

The algal symbionts were not considered when analyzing both the transcriptomic and proteomic data due to the lack of reference genomes for these diverged taxa and because there are different combinations of species present in the samples (making it challenging to reconstruct the gene inventory from the available RNA-seq data).^41^ Similarly, microbial-associated proteomics data were not included due to the challenges associated with compiling a metaproteome from reference genomes generated from unrelated environments. Traditional LC-MS/MS approaches were established to measure single species with high quality databases, such as reference genomes.^42^ We had attempted to create a database of microbial proteins, using algal transcripts constructed from the transcriptome data and reference genomes from species closely related to those present in the 16S-rRNA data, however, it was too large and highly redundant. Although, microbial and symbiont metaproteomic analysis is vital for elucidating holobiont physiological response and ability to adapt to stress, the variability in species composition across samples makes it difficult to develop robust markers of holobiont health. Therefore, we advocate for a focus on data that can be unambiguously targeted from the coral to provide a more robust platform for assessing coral stress response and development of markers of coral health.

Correlation analysis between the microbiome and metabolomic data returned very few candidate associations. This is not surprising, given the high variability observed in the microbiome and metabolome, but it does highlight the challenges associated with integrating these datasets in complex holobiont systems. It is also noteworthy that the identified associations were between metabolites with unknown structures and groups of bacteria that are poorly characterized with only very basic, general characteristic described: Coleofasciculaceae (cyanobacteria) are photosynthetic and may contribute to energy production in the coral holobiont, Patescibacteria form symbiotic associations with other organisms in the environment,^43^ and Marinibacteria are thought to participate in sulfur cycling^44^ and syntrophic degradation of amino acids.^45^ Without knowledge of metabolite structure and function, and the ecological role of each bacterial strain identified by this analysis, it is difficult to draw biologically meaningful conclusions from this analysis. This further highlights the challenges and areas where additional resources are required for coral multi-omics analysis. Additionally, given that metabolite levels are affected by the proteins encoded by the bacterial species, and not the species themselves, future studies should focus on studying shifts in the bacterial protein inventory between samples, rather than taxonomic profiles.

### Consideration with respect to experimental design

Lastly, experimental design is integral to the successful utilization of multi-omics data; whereas large samples size is generally seen as a requirement, smaller sample sizes should not be viewed as a weakness in all cases. Although the number of samples included in our analysis was small, we prioritized using nubbins from a controlled set of coral colonies (i.e., a limited set of coral genotypes) to mute the impact of genotype on multi-omics data.^46^ Furthermore, the unintentional sequencing of two samples with different genotypes demonstrates the effect of genotype on omics data, particularly proteomic data. Samples from the same genotype show limited variation in the proteome data when compared to the single sample from a different genotype (also observed by Mayfield et al.^47^). In contrast, the samples from different genotypes in the transcriptomic data often (but not always) have higher than expected variation, but this is masked by the greater overall change in these datasets. These results are consistent with the idea that different omics datasets have very different dynamics, specifically, proteomic data are under homeostatic constraints and change very little, whereas transcriptomic data are far more impacted by local environmental shifts. Additionally, given that there are practical and regulatory restrictions on the size of samples that can be collected from coral colonies, there was a limit on the number of nubbins that could be generated from each colony, and therefore how many samples from which data could be generated. This is particularly true for multi-omics studies that require all omics data to be derived from the same samples,^48^ therefore, nubbins must be large enough to allow for extraction of DNA, RNA, metabolites, and/or proteins. Small, highly controlled experiments, such as presented here with MC-289, allow for the same genotypes to be tracked across the treatments and time points, providing a useful platform to assess the different data types (i.e., free from any genotypic affects).

### Conclusion

In summary, transcriptomic and proteomic data are weakly positively correlated and provide useful (albeit, often conflicting) insights into coral biology.^49^ Metabolomics data, which assess intermediates and end products of cellular regulatory processes suffers from limited knowledge about the diversity of cnidarian metabolites and complex turnover processes (i.e., production vs. utilization). This aspect makes these results more challenging to interpret and integrate with other omics data, although stress markers which demonstrate consistent correlation with stress (e.g., dipeptides) have been identified. The usefulness of the *M. capitata* coral microbiome amplicon data is less obvious and will require coral specific databases and other types of omics analysis^50^ to provide the needed insights. Our study leads to three major conclusions about coral multi-omics data: (1) It is critical to constrain experiments with respect to genotype and treatment conditions to minimize genetic or stochastic variation in omics data. This applies particularly to the metabolomic and microbiome analyses, because these data show a more complex pattern of variation; (2) there is an urgent need for high-quality reference genomes for all members of the holobiont to facilitate analysis of meta-transcriptome and meta-genome data to elucidate biotic interactions; and (3) these experiments need to be done with multiple coral species, with dissimilarities expected in how informative the omics layers will be about fundamental processes due to differences in the underlying genetic structure, holobiont composition, and local adaptation of lineages. Accordingly, the *M. capitata* results do not capture the vast phylogenetic and metabolic diversity implicit in the term “corals”.^51^

### Limitations of the study

The results of this study are based on analysis of one or four genotypes of a single coral species under controlled conditions. Additional analysis, using different species and conditions, is needed to continue characterizing the connection between the different omics layers in corals, i.e., to determine if the patterns that we observed in a single species hold across a driver range of corals and if the magnitude of the disconnect between the omics layers is the same across species or if it varies in a predictable manner.

## STAR★Methods

### Key resources table

**Table:** 

REAGENT or RESOURCE	SOURCE	IDENTIFIER
**Critical commercial assays**		

Thermo TMT6plex	Thermo Scientific	#UF288619
SPEC Pt C18	Agilent Technologies	A57203
AllPrep DNA/RNA/miRNA Universal Kit	Qiagen	80224
TruSeq RNA Sample Preparation Kit v2	Illumina	RS-122-2001 & RS-122-2002

**Deposited data**		

Original code associated with this research	https://github.com/TimothyStephens/Montipora_capitata_multi-omics_data_correlation_comparison	Version 1
RNA-seq read data	NCBI	PRJNA694677
Proteomic data	MassIVE	MSV000088443
Microbiome 16S rRNA sequencing data	NCBI	PRJNA783340
Processed transcript, protein, 16S rRNA, and metabolite abundances	Zenodo	https://doi.org/10.5281/zenodo.6861688

**Experimental models: Organisms/strains**		

*Montipora capitata*	http://cyanophora.rutgers.edu/montipora; Shumaker et al.^19^	Version 1

**Software and algorithms**		

Proteome Discoverer 2.4	ThermoFisher	RRID:SCR_014477
mixOmics v6.18.1 R package	Rohart et al.^56^	https://www.bioconductor.org/packages/release/bioc/html/mixOmics.html; RRID:SCR_016889
El Maven	Agrawal et al.^57^	https://www.elucidata.io/el-maven; RRID:SCR_022159
Trimmomatic v0.38	Bolger et al.^58^	http://www.usadellab.org/cms/?page=trimmomatic; RRID:SCR_011848
Salmon v1.10	Patro et al.^59^	https://salmon.readthedocs.io/en/latest/index.html; RRID:SCR_017036
DESeq2 v1.34.0 R package	Love et al.^60^	https://bioconductor.org/packages/release/bioc/html/DESeq2.html; RRID:SCR_015687
Cutadapt	Martin et al.^62^	https://cutadapt.readthedocs.io/en/stable/; RRID:SCR_011841
QIIME 2 2021.4	Bolyen et al.^63^	https://qiime2.org/; RRID:SCR_008249
DADA2	Callahan et al.^64^	https://github.com/benjjneb/dada2; RRID:SCR_008205
phyloseq R package	McMurdie et al.^67^	http://www.bioconductor.org/packages/2.12/bioc/html/phyloseq.html; RRID:SCR_013080
stats R package	https://www.rdocumentation.org/packages/stats/versions/3.6.2	https://www.rdocumentation.org/packages/stats/versions/3.6.2
vegan R package	Oksanen et al.^68^	https://cran.r-project.org/web/packages/vegan/index.html; RRID:SCR_011950
psych v2.2.3 R package	Revelle et al.^69^	https://cran.r-project.org/web/packages/psych/index.html; RRID:SCR_021744
PerformanceAnalytics v2.0.4	https://cran.r-project.org/web/packages/PerformanceAnalytics/index.html	https://cran.r-project.org/web/packages/PerformanceAnalytics/index.html
eggNOG-mapper v2.1.6	Cantalapiedra et al.^71^	http://eggnog-mapper.embl.de; RRID:SCR_021165
DIAMOND v2.0.15	Buchfink et al.^73^	https://github.com/bbuchfink/diamond; RRID:SCR_016071
STAR v2.7.8a	Dobin et al.^74^	https://github.com/alexdobin/STAR; RRID:SCR_004463
Rgsam v0.1	https://github.com/djhshih/rgsam	https://github.com/djhshih/rgsam
gatk v4.2.0.0	Poplin et al.^75^	https://gatk.broadinstitute.org/hc/en-us; RRID:SCR_001876
Vcftools v0.1.17	Danecek et al.^76^	https://vcftools.github.io/index.html; RRID:SCR_001235
vcf_clone_detect.py	https://github.com/pimbongaerts/radseq	Retrieved June 12th, 2021

**Other**		

*Montipora capitata* genome data	http://cyanophora.rutgers.edu/montipora; Shumaker et al.^19^	Version 1
SILVA 16S-rRNA database	Quast et al.^65^	Release 138; RRID:SCR_006423
eggnog-mapper database	Huerta-Cepas et al.^72^	Release 2021-12-09; RRID:SCR_002456
NCBI nr database	https://ftp.ncbi.nlm.nih.gov/blast/db/	Release 2022_07

### Resource availability

#### Lead contact

Further information and requests for resources and reagents should be directed to and will be fulfilled by the lead contact, Timothy Stephens (ts942@sebs.rutgers.edu).

#### Materials availability

This study did not generate new unique reagents.

### Experimental model and study participant details

Four colonies of *M. capitata* coral collected from Kāne‘ohe Bay, HI.

### Method details

#### Overview of experimental design

The methods for *M. capitata* colony collection, cultivation, and the design of the heat stress experiment are described in Williams et al*.*^18^ Briefly, four colonies (genotypes) of *M. capitata* (designated genotypes MC-206, MC-248, MC-289, and MC-291) were collected from Kāne‘ohe Bay, HI, (under SAP 2019-60), and fragmented into 30 pieces before being fixed to labeled plugs using hot-glue. The 30 nubbins from each genotype were randomly distributed across tanks that were supplied with a steady flow of water directly from the bay. The temperature of the tanks was controlled by heaters and lights were used to simulate a 12-hour light/12-hour dark cycle. The nubbins were left to acclimate at ambient temperature (∼27°C) for 5 days before the high-temperature treatment tanks were increased by ∼0.4°C every 2 days for a total of 9 days, until they were between 30.5-31.0°C. The treatment (hereinafter, high temperature) tanks were held at ∼30.5°C and the control (hereinafter, ambient temperature) tanks at ∼ 27.5°C until the end of the experiment, which lasted an additional 16 days. The temperature of 30.5°C was chosen for thermal stress because it is the expected range for natural warming events in Kāne‘ohe Bay.^52^ Three nubbins per genotype (n=3 replicates) were collected at five time points (TP1-5) during the experiment, however, only samples from TP1 (after temperature ramp-up was complete), TP3 (at the onset of bleaching; 13 days after TP1), and TP5 (on last day of the experimental period; 17 days after TP1) were processed for multi-omics analysis. Bleaching progression was monitored using color scores^53^ generated for the ambient and stress treated nubbins at each of the five time points (Figure S1).^18^ The samples collected at each time point were flash-frozen in liquid N_2_ and stored at –80°C; each frozen sample was divided into subsamples which were processed for a different multi-omics method. Three nubbins were also collected from the same coral colonies in Kāne‘ohe Bay (hereinafter, field samples), flash frozen in liquid N_2_, and stored at –80°C. These frozen field samples were processed for multi-omics using the same protocols applied to the time-point samples. Samples from all four colonies/genotypes were used for metabolomics (published in Williams et al.^18^) and 16S-rRNA microbiome (published in this study) analysis; samples from colony MC-289 were used for RNA sequencing (published in Williams et al.^20^) and proteomic analysis (published in this study). The choice to focus on just a single genotype for RNA sequencing and proteomic analysis was based on resource constraints associated with the project.

#### Proteomic data

Proteomic data were generated for MC-289 from two out of the three replicate nubbins per time point (TP1, TP3, and TP5) per condition (including field samples). The proteins were extracted using a protocol adapted from Stuhr et al.^54^ The lysis buffer comprised 50 mM Tris-HCl (pH 7.8), 150 mM NaCl, 1% SDS, and cOmplete, Mini EDTA-free Tablets. One gram of each sample was ground in a mortar on ice with 100 μl of lysis buffer. The sample was then transferred to a 2mL Eppendorf tube, with an additional 50 μl of lysis buffer used to wash the mortar, for a total lysate volume of 150 μl. Each sample was vortexed for 1 min, stored on ice for 30 min, and clarified by centrifugation at 10,000 rcf for 10 min (4°C). Protein concentrations were measured using the Pierce 660-nm Protein Assay. Thereafter, 40 μg of each sample was run on an SDS-PAGE gel, with slices collected and incubated at 60°C for 30 min in 10 mM Dithiothreitol (DTT). After cooling to room temperature, 20 mM iodoacetamide was added to the gel slices before they were kept in the dark for 1 hour to block free cysteine. The samples were digested using trypsin at a concentration of 1:50 (w:w, trypsin:sample) before being incubated at 37°C overnight. The digested peptides were dried under vacuum and washed with 50% acetonitrile to pH neutral. The digested peptides were labeled with Thermo TMT6plex (Lot #: UF288619) following the manufacturer’s protocol, before being pooled together at a 1:1 ratio. The pooled samples were dried and desalted with SPEC Pt C18 (Agilent Technologies, A57203) before fractionation using an Agilent 1100 series machine. The samples were solubilized in 250 μl of 20 mM ammonium (pH10), and injected onto an Xbridge column (Waters, C18 3.5 μm 2.1X150 mm) using a linear gradient of 1% buffer B/min from 2-45% of buffer B (B: 20 mM ammonium in 90% acetonitrile, pH10). UV 214 was monitored while fractions were collected. Each fraction was desalted^55^ and analyzed by LC-MS/MS.

Nano-LC-MSMS was performed using a Dionex rapid-separation liquid chromatography system interfaced with an Eclipse (Thermo Fisher Scientific). Selected desalted fractions 28-45 were loaded onto an Acclaim PepMap 100 trap column (75 μm x 2 cm, ThermoFisher) and washed with 0.1% trifluoroacetic acid for 5 min with a flow rate of 5 μl/min. The trap was brought in-line with the nano analytical column (nanoEase, MZ peptide BEH C18, 130A, 1.7μm, 75μm x 20cm, water) with a flow rate of 300 nL/min using a multistep gradient: 4% to 15% of 0.16% formic acid and 80% acetonitrile in 20 min, then 15%–25% of the same buffer in 40 min, followed by 25%–50% of the buffer in 30 min. The scan sequence began with an MS1 spectrum (Orbitrap analysis, resolution 120,000, scan range from 350–1600 Th, automatic gain control (AGC) target 1E6, maximum injection time 100 ms). For SPS3, MSMS analysis consisted of collision-induced dissociation (CID), quadrupole ion trap analysis, automatic gain control (AGC) 2E4, NCE (normalized collision energy) 35, maximum injection time 55ms, and isolation window at 0.7. Following acquisition of each MS2 spectrum, we collected an MS3 spectrum in which 10 MS2 fragment ions are captured in the MS3 precursor population using isolation waveforms with multiple frequency notches. MS3 precursors were fragmented by HCD and analyzed using the Orbitrap (NCE 55, AGC 1.5E5, maximum injection time 150 ms, resolution was 50,000 at 400 Th scan range 100-500). The whole cycle is repeated for 3 seconds before repeating from an MS1 spectrum. Dynamic exclusion of 1 repeat and duration of 60 sec was used to reduce the repeat sampling of peptides. LC-MSMS data were analyzed with Proteome Discoverer 2.4 (ThermoFisher) with the sequence search engine run against the protein sequences of the genes predicted in the published *M. capitata* genome^19^ and a database that consisted of common lab contaminants. The MS mass tolerance was set at ± 10 ppm, MSMS mass tolerance was set at ± 0.4 Da for the proteome. TMTpro on C and N-terminus of peptides and carbamiodomethyl on cysteine was set as static modification. Methionine oxidation, protein N-terminal acetylation, protein N-terminal methionine loss or protein N-terminal methionine loss plus acetylation were set as dynamic modifications for proteome data. Percolator was used for results validation. Concatenated reverse database was used for the target-decoy strategy.

For reporter ion quantification, the reporter abundance was set to use the signal/noise ratio (S/N) only if all spectrum files had S/N values, otherwise, intensities were used instead of S/N values. The quant value was corrected for isotopic impurity of reporter ions. Co-isolation threshold was set at 50%. The average reporter S/N threshold was set to 10 and the SPS mass matches percent threshold was set to 65%. The protein abundance of each channel was calculated using summed S/N of all unique and razor peptides. Finally, the abundance was further normalized to a summed abundance value for each channel over all peptides identified within a file (https://zenodo.org/record/6861688). Only peptide sequences from genes predicted in the *M. capitata* genome were used in this study. Proteins with a false discovery rate (FDR) < 0.01 and were considered “high” confidence, and proteins with a FDR ≥ 0.01 but < 0.05 were considered “medium” confidence. Differentially expressed proteins (DEPs; *p*-value < 0.05) were identified between the ambient and high temperature treatments for each time point using the stats v4.1.2 and mixOmics v6.18.1^56^ R packages. Adjusted *p*-values were not used for this analysis due to the low number of replicates per condition.

#### Polar metabolomic data

Polar metabolite data were generated for each of the four genotypes across the three analyzed time points, two treatment conditions, and field colonies (n=3). The methods used for polar metabolite extraction and analysis are described in Williams et al*.*^18^ Briefly, metabolites were extracted from each sample with a 40:40:20 (MeOH: ACN: H_2_O + Formic Acid) extraction buffer and followed a protocol optimized for the extraction of water-soluble polar metabolites; the resulting metabolite extracts were separated into phases using hydrophilic interaction liquid chromatography (performed on a Vanquish Horizon UHPLC system). A Thermo Fisher Scientific Q Exactive Plus was used for the full-scan MS analysis and to generate the MS^2^ spectra. The resulting metabolite data was analyzed using El Maven.^57^ Peaks that had ion counts above 50,000 (before normalization) were retained. The metabolite profiles for all samples were aligned using OBI-Warp. The metabolite intensities were normalized using the frozen weights of each sample (https://zenodo.org/record/6861688). Metabolites in the resulting list were filtered, retaining only those with 48 good peaks and a maxQuality score of 0.8, and a total ion count of ≥ 1000 across all samples. These filtered and normalized peaks were used for downstream analysis and to generate total metabolite counts.

#### Transcriptomic data

RNA-seq data was generated for MC-289 across the three analyzed time points, two treatment conditions, and field colony (n=3). The methods for cDNA library preparation, sequencing and data analysis are detailed in Williams et al*.*^20^ Briefly, a Qiagen AllPrep DNA/RNA/miRNA Universal Kit was used to extract RNA from the (crushed) frozen samples; a TruSeq RNA Sample Preparation Kit v2 was used to generate strand specific cDNA libraries that were sequenced on a NovaSeq (2x150 bp) machine. This protocol included a poly-A selection step, which enriched for transcripts from eukaryotic cells and depleted those from the prokaryotic microbiome. RNA-seq reads were trimmed for low quality bases and adapters using Trimmomatic v0.38;^58^ read pairs where both mates survived trimming were used to quantify (using Salmon v1.10^59^) the expression levels of the genes predicted in the *M. capitata* genome^19^ (https://zenodo.org/record/6861688). Differentially expressed genes (DEGs; adjusted *p*-value < 0.05) were identified between the ambient and high temperature treatments at each time point by the DESeq2 v1.34.0^60^ R package using the aligned read counts produced by Salmon. The Transcripts Per Million (TPM) normalized expression values produced by Salmon were used for all downstream visualization and ordination analyses. Transcripts with a cumulative TPM > 100 (i.e., > 100 TPM summed across all samples) were used for the ordination and statistical analysis described below.

#### Microbiome data

Microbiome V3-V4 hypervariable region 16S-rRNA sequencing data were generated for each of the four genotypes across the three analyzed time points, two treatment conditions, and for the field colonies (n=3). The cells in each sample were lysed using liquid nitrogen and mechanical grinding. Total DNA was isolated using a Qiagen AllPrep DNA/RNA/miRNA Universal Kit, following the manufacturer’s instructions (Table S6). The 16S-rRNA amplicon sequencing libraries were prepared as per Illumina’s instructions,^61^ using primers designed for the V3 and V4 hypervariable region (Forward Primer: 5'-TCGTCGGCAGCGTCAGATGTGTATAAGAGACAGCCTACGGGNGGCWGCAG; Reverse Primer: 5'-GTCTCGTGGGCTCGGAGATGTGTATAAGAGACAGGACTACHVGGGTATCTAATCC), the Nextera XT library preparation kit, and dual indexes (i7 and i5). The PCR mix and thermocycler conditions are listed in Illumina’s instructions.^61^ A negative control was run along with the samples to ensure there was no contamination in the PCR mix. The libraries were pooled together and a 20% PhiX spike-in was added. Quality control was performed using a Qubit fluorometer and an Agilent Bioanalyzer, with the target library length being ∼600 bp. Libraries were sequenced by Genewiz on an Illumina MiSeq (2x300 bp) machine (Table S7). Raw reads were trimmed for quality and removal of primer sequence using Cutadapt.^62^ Quality trimming and filtering, denoising, merging and chimera removal, and amplicon sequence variant [ASV] feature table construction were carried out using the QIIME 2 2021.4^63^ plug-in for DADA2.^64^ Taxonomic assignment was carried out with QIIME2 against the SILVA 16S-rRNA database (release 138).^65^ The initial ASV feature table derived from the trimmed reads (https://zenodo.org/record/6861688) was filtered, removing ASV that were: (1) too short (< 390 bases); (2) did not have unambiguous taxonomic assignments to at least the phylum-level; (3) had taxonomic assignments of “Archaea”, “Chloroplast” or “Mitochondria”; and (4) had a frequency of < 20 reads across all 83 samples (Table S8; https://zenodo.org/record/6861688). The < 20 reads cutoff was chosen based on similar filtering approaches deployed in other studies.^66^ These studies, which often consisted of < 30 samples, used cutoffs of < 10 reads, therefore, to account for the larger number of samples in our analysis, a cutoff of 20 reads was chosen. Per-sample ASV counts were rarefied prior to α- and β-diversity analysis. Shapiro-Wilk tests of Shannon and Simpson α-diversity metrics show that the data are non-normal (*p*-value < 0.01). Analyses of α- and β-diversity were carried out in R using the phyloseq,^67^ stats, and vegan^68^ packages. To visualize β-diversity, samples were rarefied to 39,902 reads per sample (chosen by rarefaction analysis to minimize loss of data) using the *rarefy_even_depth* function in the phyloseq^67^ R package, after which the Bray-Curtis distances between samples were calculated using the *distance* function.

The psych v2.2.3^69^ R package was used to determine whether significant correlations exist between the 16S-rRNA amplicon and metabolite data. Amplicon count data were agglomerated by taxon at multiple taxonomic ranks—from phylum to genus—for testing using the *tax_glom* function within the phyloseq package. Pairwise correlations using the Spearman method, as well as adjustment of *p*-values using the Benjamini-Hochberg method, were performed on both raw and normalized (by relative abundance) quantifications using the *corr.test* function. Correlations were retained for further analysis if the associated adjusted *p*-value was less than 0.05. Furthermore, because Spearman rank correlation analyses are sensitive to low values, only taxa with greater than 200 observations across all samples were considered. Correlations were visualized with correlation plots and histograms, generated using the *chart* function in the PerformanceAnalytics v2.0.4^70^ R package.

#### Gene functional annotation

Functional assignment of the *M. capitata* proteins was done using eggNOG-mapper (v2.1.6; --pfam_realign denovo; database release 2021-12-09)^71^^,^^72^ and a DIAMOND search (v2.0.15; blastp --ultra-sensitive --max-target-seqs 1000)^73^ against the NCBI nr database (release 2022_07). eggNOG-mapper was also used to assign KEGG orthologous numbers (KO numbers).

#### Proportion of shared SNPs between transcriptome samples

The proportion of single nucleotide polymorphisms (SNPs) shared between each pairwise combination of transcriptome samples was used to confirm that they were all derived from the same colony (genotype). Each sample was aligned against the *M. capitata* reference genome^19^ using STAR (v2.7.8a; --sjdbOverhang 149 --twopassMode Basic).^74^ Read-group information was extracted from the read names using rgsam (v0.1; https://github.com/djhshih/rgsam; --qnformat illumina-1.8) and added to the aligned reads using gatk FastqToSam and gatk MergeBamAlignment (--INCLUDE_SECONDARY_ALIGNMENTS false --VALIDATION_STRINGENCY SILENT). Duplicate reads were removed using gatk MarkDuplicates (--VALIDATION_STRINGENCY SILENT) before reads that spanned intron-exon boundaries were split using gatk SplitNCigarReads (default). Haplotypes were called using gatk HaplotypeCaller (-dont-use-soft-clipped-bases -ERC GVCF), with the resulting GVCF files (one per sample) combined using gatk CombineGVCFs before being jointly genotyped using gatk GenotypeGVCFs (-stand-call-conf 30).^75^ The resulting variant were filtered for indels, sites with low average reads coverage across all samples, and sites without called genotypes across all samples using vcftools (v0.1.17; --remove-indels --min-meanDP 10 --max-missing 1.0).^76^ The “vcf_clone_detect.py” script (from https://github.com/pimbongaerts/radseq; retrieved June 12^th^, 2021) was used with the filtered variants to compute the number of SNPs shared between each pair of samples.

#### Correlation between gene expression and protein abundance

The correlation between gene expression and protein abundance was assessed using the samples from MC-289. Only genes (n=4036) which were detected in the proteome data of at least one sample were used in this analysis (Table S1). The TPM-normalized gene expression and abundance-normalized protein counts were scaled using a log_2_ transformation (with an offset of 1 to prevent infinite log values) before being plotted. The magnitude and directionality of the stress response of the genes detected in the proteome data was assessed using the log_2_ FC values computed during differential transcriptome and proteome expression analysis.

#### PCA, PCoA, and PERMANOVA

Principal component analysis (PCA), principal coordinate analysis (PCoA), and permutational multivariate ANOVA (PERMANOVA) were performed on the normalized metabolomic, transcriptomic (cumulative TPM > 100), proteomic, and 16S microbiome datasets. PCA was done using the *prcomp* function (center = FALSE, scale = FALSE) from the stats v4.1.2 R package. PERMANOVA tests were conducted on Bray-Curtis dissimilarity matrices using the *adonis2* (permutations = 999, method = 'bray'; using replicate tank as the strata) and *vegdist* (method = 'bray') functions in the vegan v2.6-268 R package. Only 190 permutations were used for the proteomics PERMANOVA as this was the maximum value possible given the smaller number of samples in the dataset. When analyzing just the MC-289 samples, the PERMANOVA formula “TimePoint ∗ Treatment” was used, when analyzing all genotypes, the formula “TimePoint ∗ Treatment ∗ Genotype” was used. It should be noted that the most significant *p*-value produced by this analysis is *p* = 0.001. PCoA was performed on the Bray-Curtis dissimilarity matrix using *wcmdscale* function (k = 2, eig = TRUE, add = "cailliez") from the vegan v2.6-5^68^ R package.

#### Sparse PLS-DA

The machine-learning method, partial least squares-discriminant analysis (PLS-DA), was performed on the normalized metabolomic, transcriptomic (cumulative TPM > 100), and proteomic datasets using the *splsda* function (ncomp = 6, scale = FALSE, near.zero.var = TRUE) from the mixOmics v6.18.1^56^ R package. For the 16S microbiome dataset, the *perf* function was used to evaluate the performance of PLS-DA using repeated *k*-fold cross-validation (validation = "Mfold", nrepeat = 50). The *tune* function was then applied to determine the number of variables (1-1000) to select on each component for sparse PLS-DA (dist = 'max.dist', measure = "BER", nrepeat = 50), before the *splsda* function was run with the tuned number of components and variables per component.

### Quantification and statistical analysis

The association between experimental factors and the variance in each of the omics datasets were assessed using permutational multivariate ANOVA (PERMANOVA) analysis run using 999 permutations (except for the proteomic dataset which used 190 permutations due to the small number of samples in the dataset). Three replicate samples were available per condition, per time point, per genotype for the transcriptome, metabolome, and microbiome datasets; two replicate samples were available per condition, per time point, per genotype for the proteome dataset. The results from this analysis are presented in Tables S3 and S4.

## Data Availability

• The metabolomic data used in this study were generated and preprocessed by Williams et al.^18^ and is available as Supplementary Data files associated with that publication. The RNA-seq read data used in this study were generated and preprocessed by Williams et al.^20^ and are available under NCBI BioProject ID: PRJNA694677. The proteomic data generated by this study are available from MassIVE under the ID MSV000088443. The microbiome 16S rRNA sequencing data generated by this study are available under NCBI BioProject ID PRJNA783340. Processed transcript, protein, 16S rRNA, and metabolite abundances are available as supplemental datasets from Zenodo (https://doi.org/10.5281/zenodo.6861688). • All original code has been deposited at GitHub and is publicly available as of the date of publication. The link is listed in the key resources table. • Any additional information required to reanalyze the data reported in this paper is available from the lead contact upon request.

## Supplemental information

Document S1. Figures S1–S10

Table S1. Protein and transcript FC values for genes identified in proteomic data, related to Figure 2 The 138 stress-response related genes are also highlighted.

Table S2. Major KEGG pathways used to filter proteins identified in the proteomic data, related to Figure 2

Table S3. Results from the PERMANOVA tests run on the transcriptomic, proteomic, and metabolomic datasets, related to Figures 1 and 3

Table S4. Results from the statistical tests run on the alpha- and beta-diversity metrics, related to Figure 4

Table S5. Abundance of bacterial phyla across the processed 16S rRNA data from the four *M. capitata* colonies (n = 3 per treatment/time point/colony), related to Figure 4

Table S6. Extraction yield of DNA (and for MC-289, RNA) from the four *M. capitata* colonies (n = 3 per treatment/time point/colony), related to Figures 1, 2, and 4

Table S7. Read statistics for the microbiome 16S rRNA data from the four *M. capitata* colonies (n = 3 per treatment/time point/colony), related to Figure 4

Table S8. Number of reads discarded during the 16S rRNA read processing workflow, related to Figure 4
