# Supplementary material for: Peeling back the layers of coral holobiont multi-omics data
Source: iScience. 2023 Aug 14;26(9):107623. doi: 10.1016/j.isci.2023.107623 (PMC10482995; doi:10.1016/j.isci.2023.107623)
Supplement: Document S1. Figures S1–S10 [file mmc1.pdf]

iScience, Volume *26*

Supplemental information

Peeling back the layers of coral holobiont multi-omics data

Amanda Williams, Timothy G. Stephens, Alexander Shumaker, and Debashish Bhattacharya

Supplemental Figures

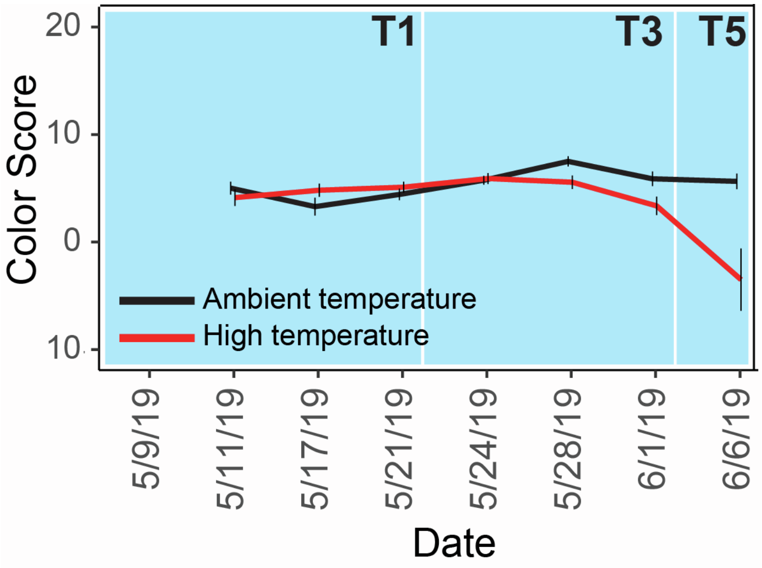

Figure S1. Average color scores of *M. capitata* coral nubbins under ambient (black) and high (red) temperature treatment conditions over the course of the experiment. Error bars are shown for the color scores observed at each time point. The time points (T1, T3, and T5) when samples were collected for omics data generation are labeled. Related to Figures 1, 2, 3, and 4.

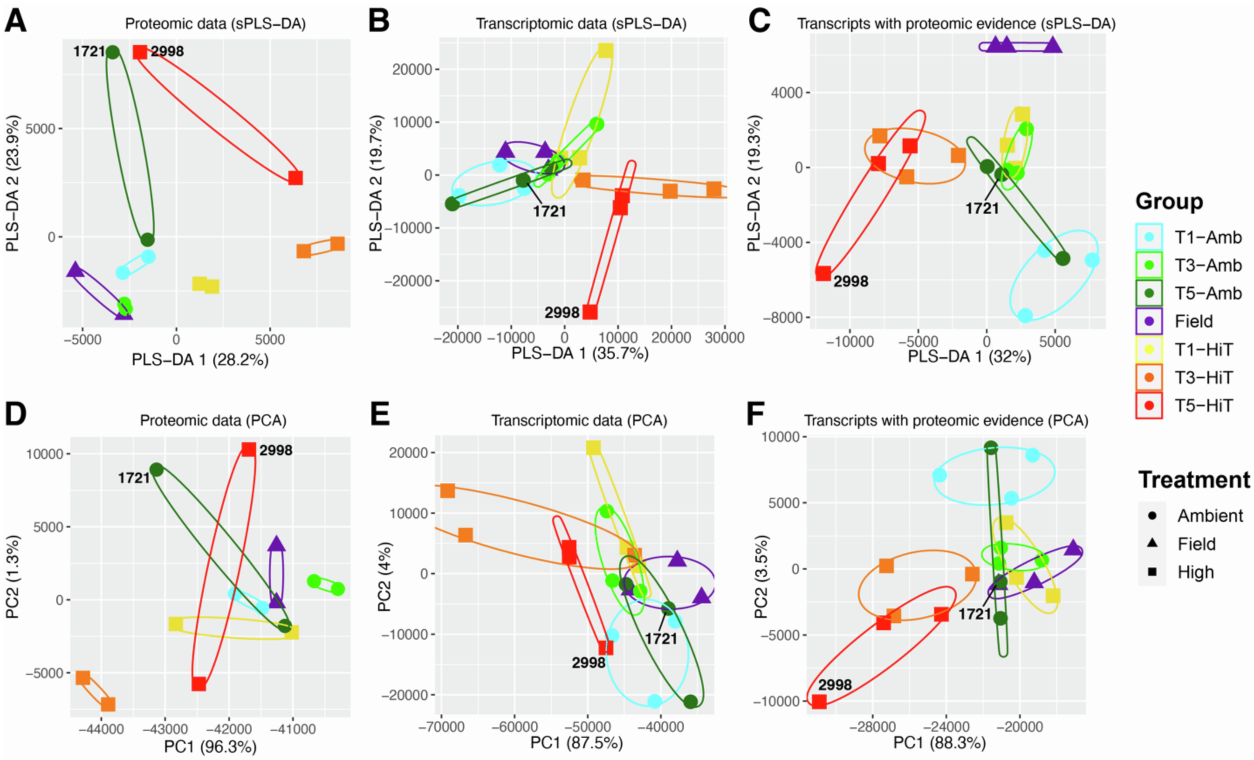

Figure S2. Relationship between proteomic and transcriptomic samples from one genotype (MC-289) of *M. capitata*. (A, B, C) sPLS-DA and (D, E, F) PCA plots generated using the proteomic data, transcriptomic data, and transcripts with proteomic evidence, respectively. The shape of each point corresponds to the treatment (ambient, high temperature, or field samples) and the color corresponds to the treatment and time point at which each sample was collected; a legend with this information is displayed on the right side of the image. Samples from the same condition are grouped with colored ellipses. The amount of variance explained by each axis in each plot is displayed in parentheses. Samples derived from mislabeled genotypes are annotated with their respective plug IDs (2998 for MC-289_T5-HiT_2998 and 1721 for MC-289_T5-Amb_1721). Related to Figure 1.

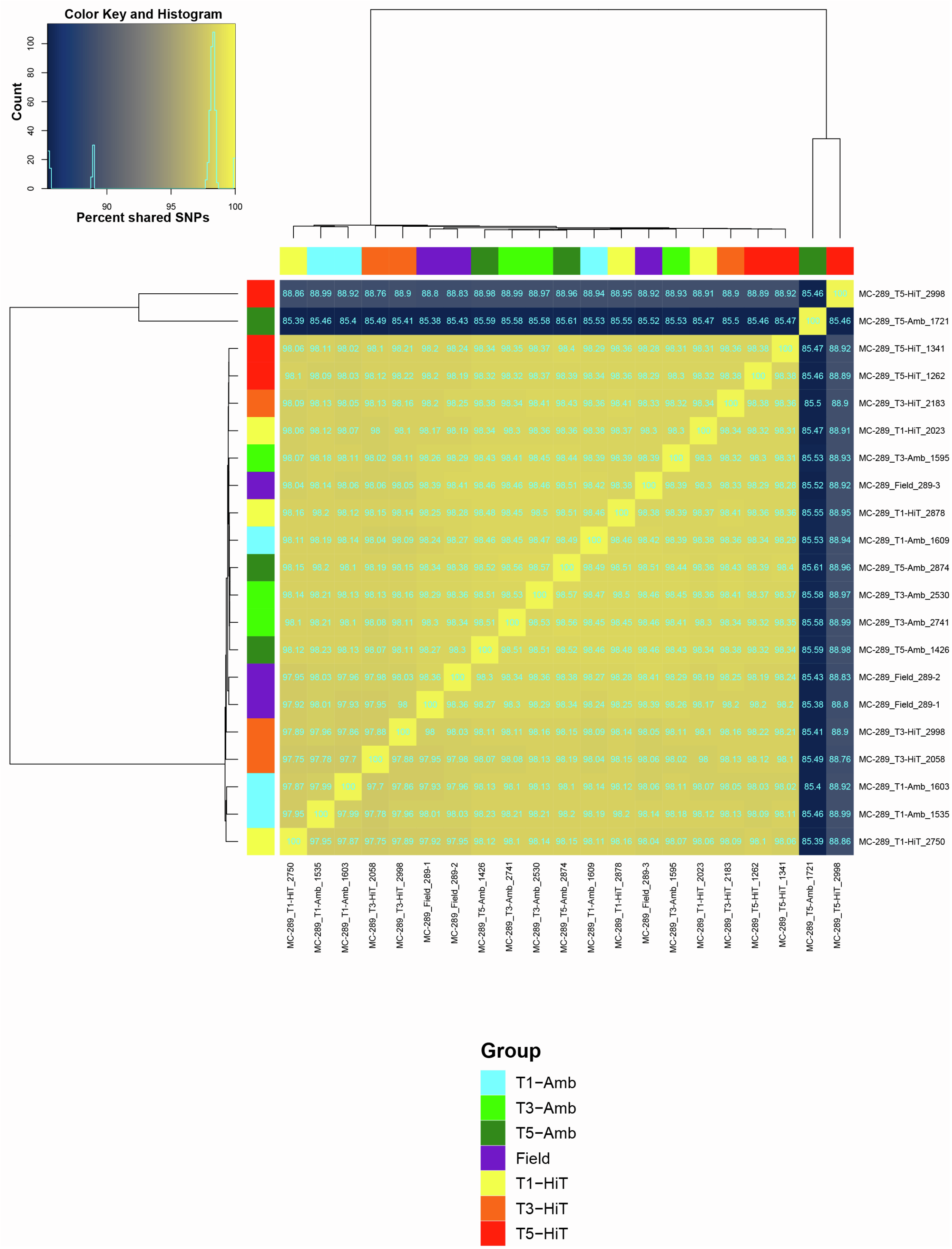

Figure S3. Proportion of SNPs shared between each of the MC-289 transcriptome samples. Heatmap showing the proportion of shared SNPs between all pair-wise combinations of the MC-289 transcriptome

samples. The value of each pairwise combination is shown on the heatmap. A histogram showing the number of pairwise comparisons with a given proportion of shared SNPs is shown in the top left of the figure; the background colors used in the histogram correspond to the colors used in the heatmap. A legend describing the colors used along the top and left sides of the heatmap is presented at the bottom of the figure. The order of the columns and rows, and the dendrograms presented on the top and left sides of the heatmap, were generated by hierarchical clustering of the proportion of shared SNPs between each of the samples. Related to Figures 1 and 2.

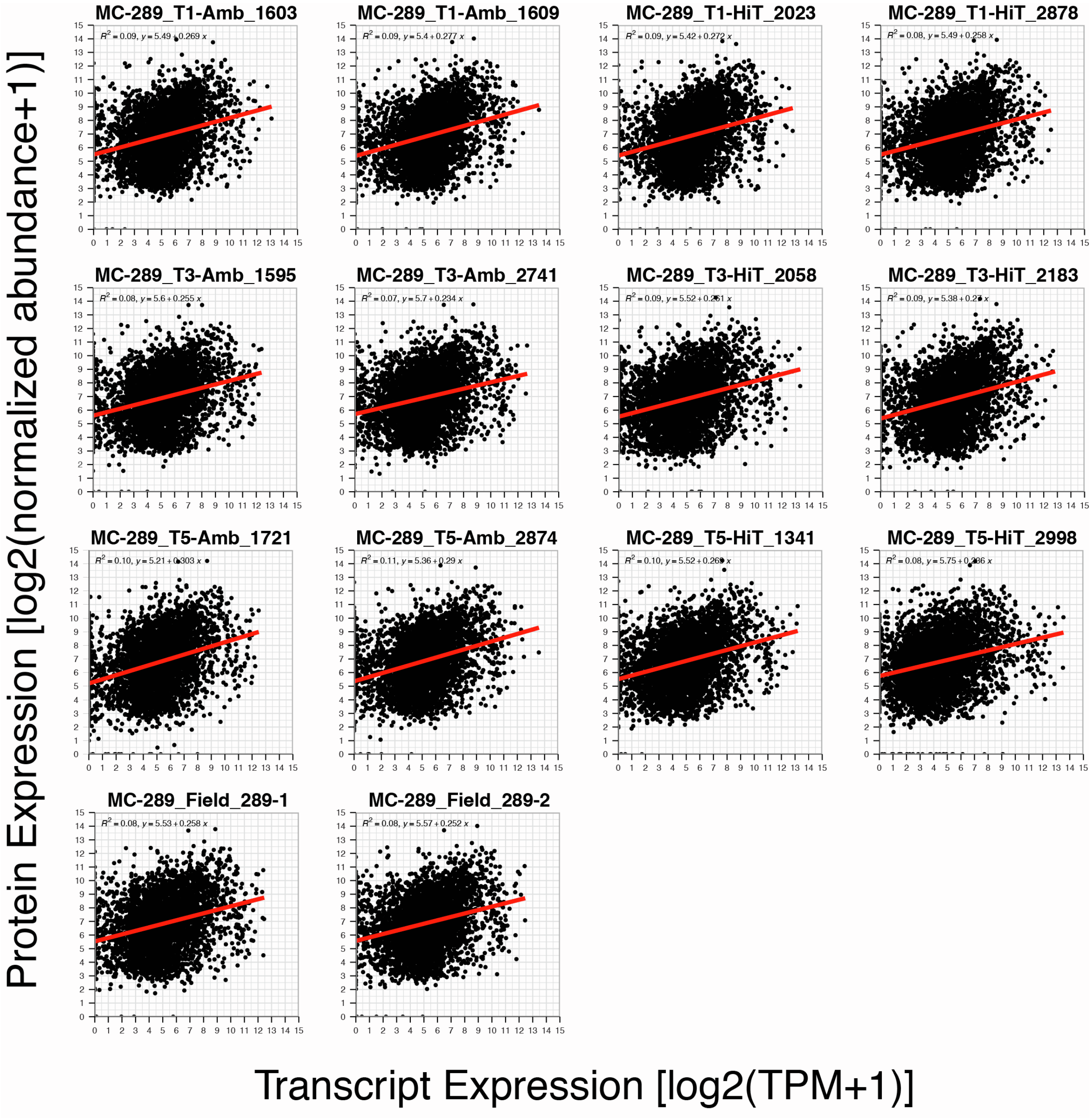

Figure S4. Correlation between the $\log_2$ transformed transcript and protein expression values of the genes detected in the proteomic data (n = 4036) from each treatment group. A trend line (red) is fitted through the data with associated R^2 value and line formula shown. Related to Figures 1 and 2.

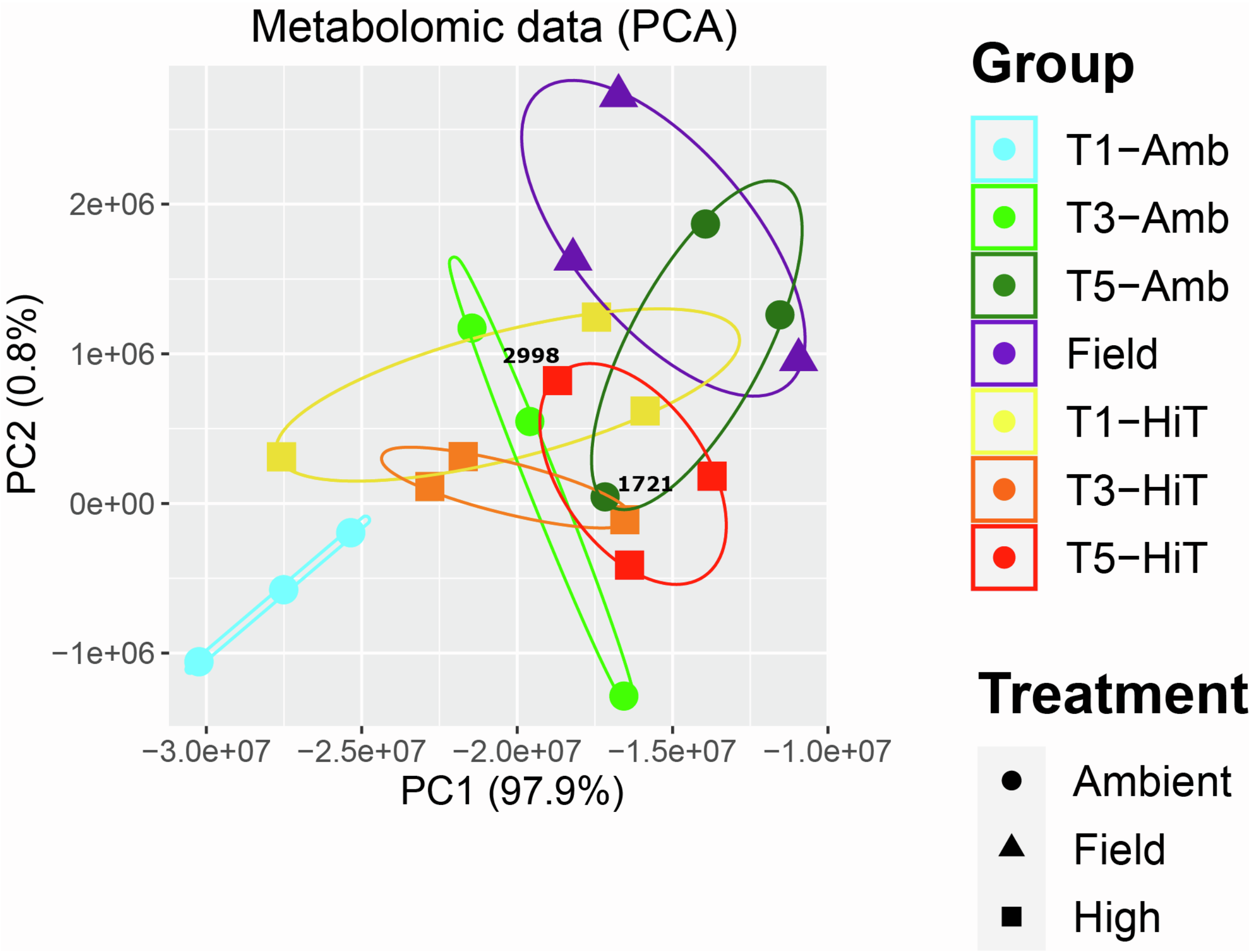

Figure S5. Relationship between metabolomic samples from one genotype (MC-289) of *M. capitata* presented as a PCA plot. The shape of each point corresponds to the treatment (i.e., ambient, high temperature, or field) and the color corresponds to the treatment and time point at which each sample was collected; a legend with this information is displayed on the right side of the image. Samples from the same condition are grouped with colored ellipses. The amount of variance explained by each axis in each plot is displayed in parentheses. Samples derived from mislabeled genotypes are annotated with their respective plug IDs (2998 for MC-289_T5-HiT_2998 and 1721 for MC-289_T5-Amb_1721). Related to Figure 3.

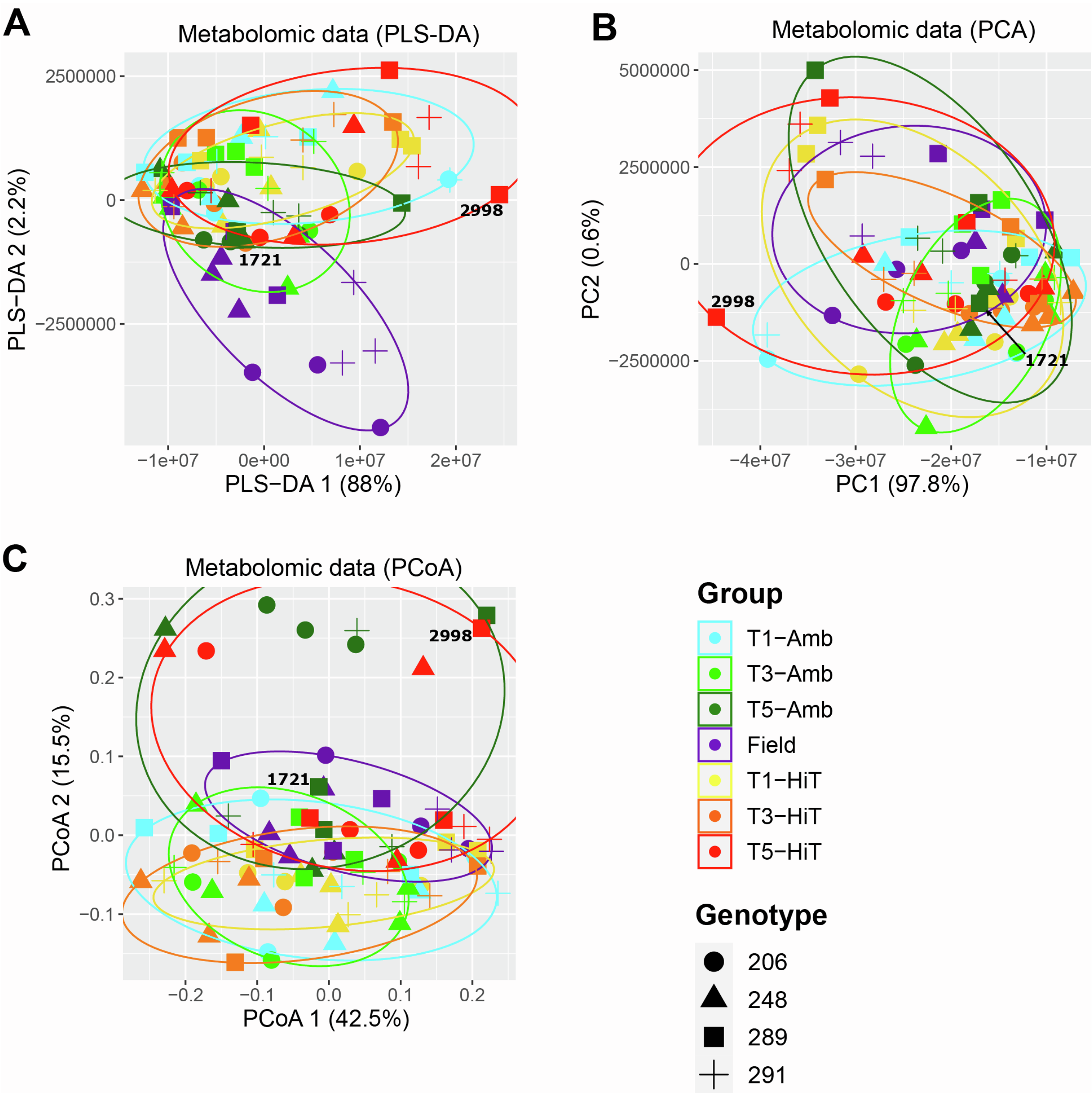

Figure S6. Relationship between metabolomic samples from four genotypes of *M. capitata* presented as (A) sPLS-DA, (B) PCA, and (C) PCoA plots. PCoA plots are based on Bray-Curtis distances between all samples in the corresponding dataset. The shape of each point corresponds to the genotype and the color corresponds to the treatment and time point at which each sample was collected; a legend with this information is displayed in the bottom right corner of the image. Samples from the same condition are grouped with colored ellipses. The amount of variance explained by each axis in each plot is displayed in parentheses. Samples derived from mislabeled genotypes are annotated with their respective plug IDs (2998 for MC-289_T5-HiT_2998 and 1721 for MC-289_T5-Amb_1721). Related to Figure 3.

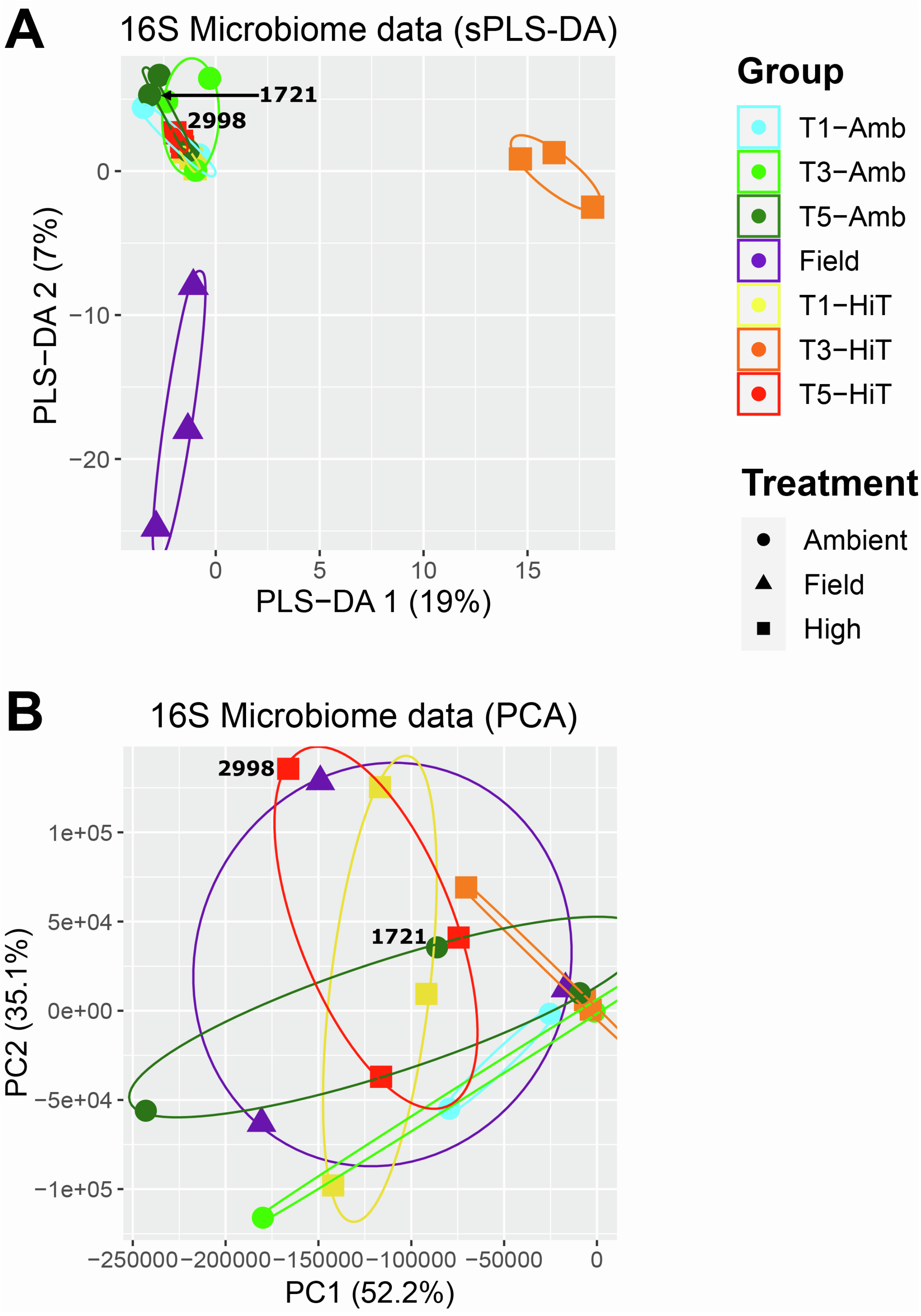
A
16S Microbiome data (sPLS-DA)
1721
2998
PLS−DA 2 (7%)
PLS−DA 1 (19%)
0
−10
−20
0
5
10
15
Group
T1−Amb
T3−Amb
T5−Amb
Field
T1−HiT
T3−HiT
T5−HiT
Treatment
Ambient
Field
High
B
16S Microbiome data (PCA)
2998
1721
PC2 (35.1%)
PC1 (52.2%)
1e+05
5e+04
0e+00
−5e+04
−1e+05
−250000
−200000
−150000
−100000
−50000
0

Figure S7. Relationship between microbiome samples from one genotype (MC-289) of *M. capitata* presented as (A) PLS-DA and (B) PCA plots. The color of each point corresponds to the treatment and time point at which each sample was collected; a legend with this information is displayed on the right of the image. Samples from the same condition are grouped with colored ellipses. The amount of variance explained by each axis in each plot is displayed in parentheses. Samples derived from mislabeled genotypes are annotated with their respective plug IDs (2998 for MC-289_T5-HiT_2998 and 1721 for MC-289_T5-Amb_1721). Related to Figure 4.

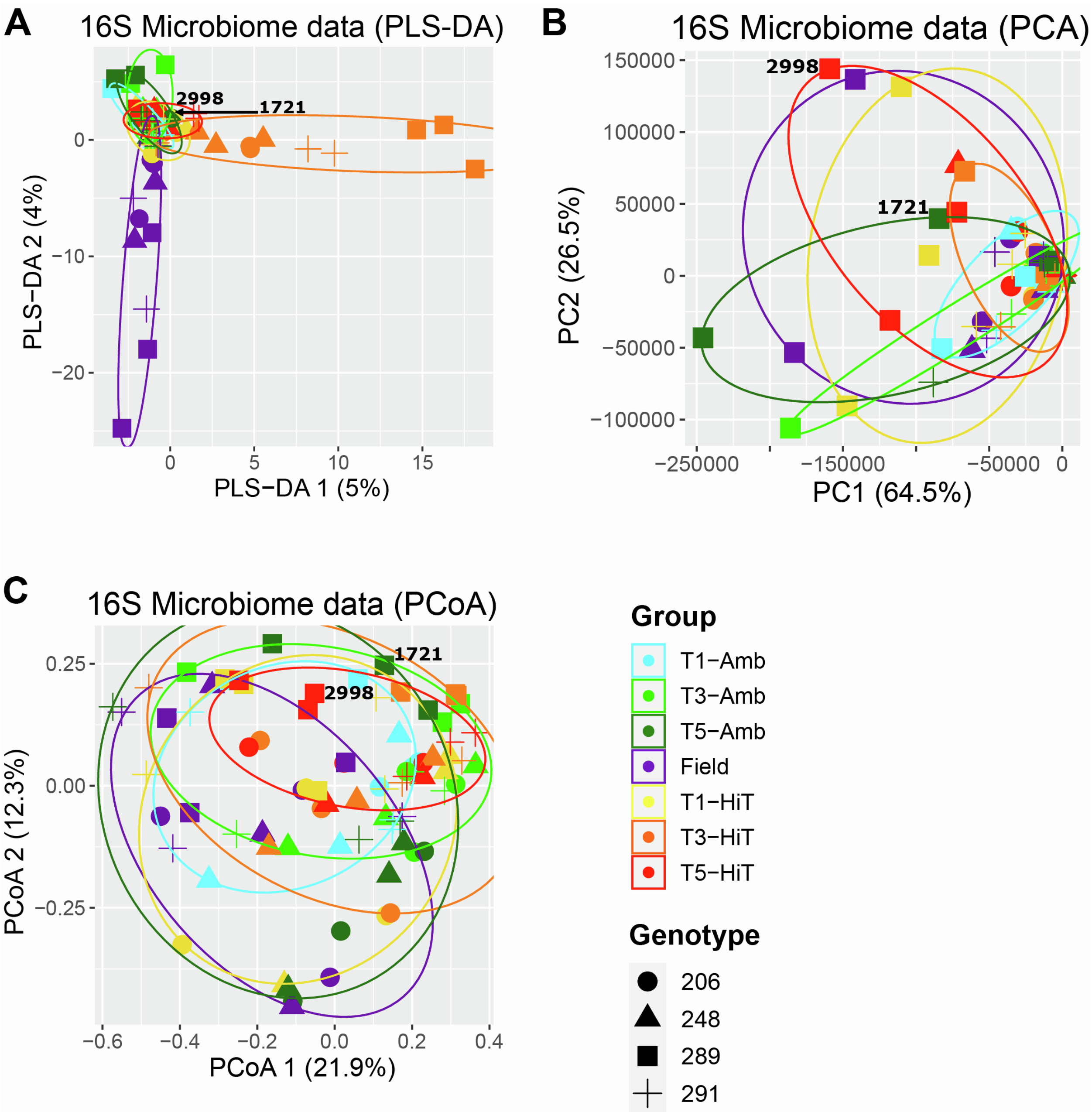

Figure S8. Relationship between microbiome samples from four genotypes of *M. capitata* presented as (A) PLS-DA, (B) PCA, and (C) PCoA plots. PCoA plots are based on the Bray-Curtis distances between all samples in the corresponding dataset. The shape of each point corresponds to the genotype and the color corresponds to the treatment and time point at which each sample was collected; a legend with this information is displayed in the bottom right corner of the image. Samples from the same condition are grouped with colored ellipses. The amount of variance explained by each axis in each plot is displayed in parentheses. Samples derived from mislabeled genotypes are annotated with their respective plug IDs (2998 for MC-289_T5-HiT_2998 and 1721 for MC-289_T5-Amb_1721). Related to Figure 4.

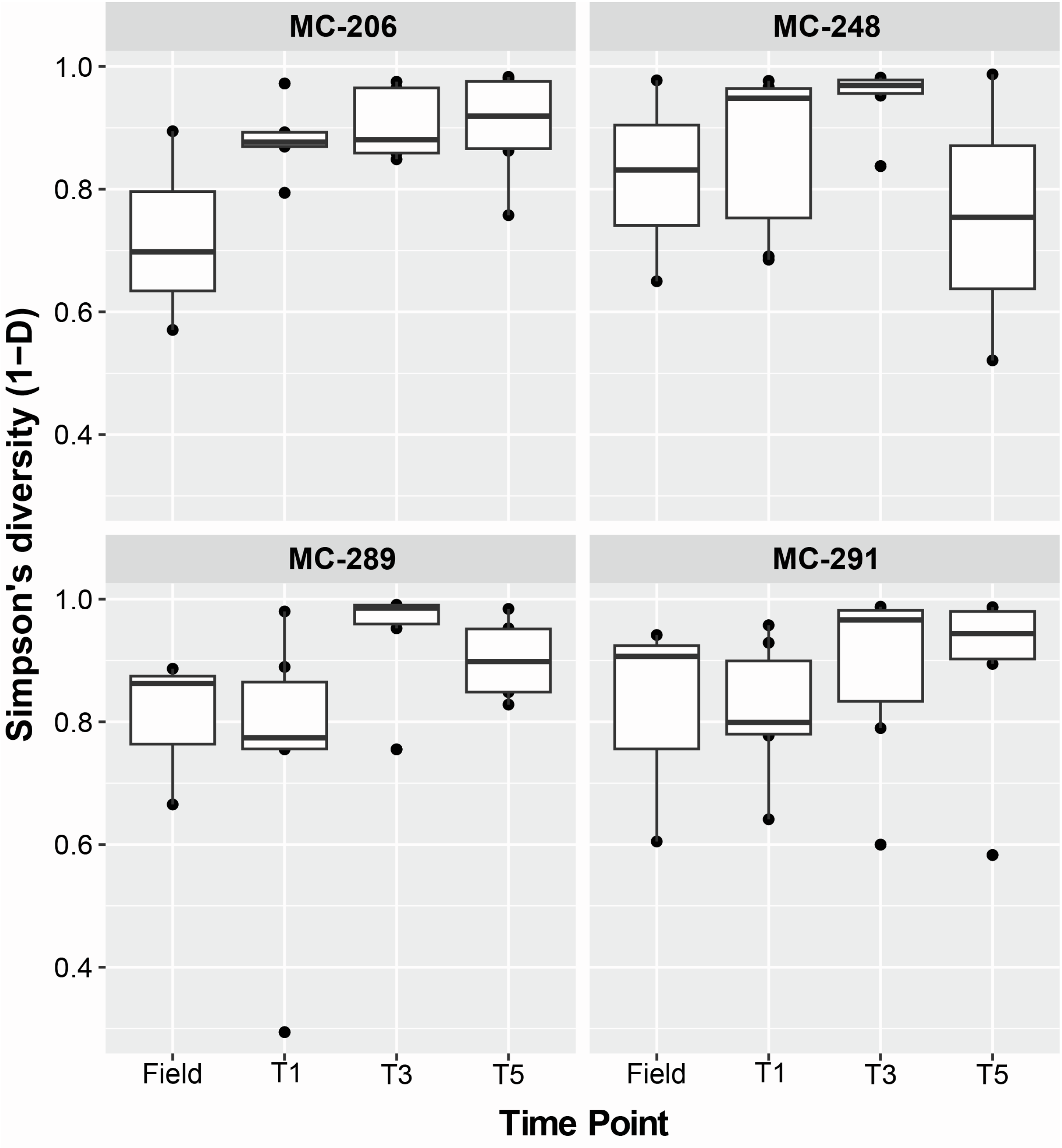

Figure S9. Change in 16S microbiome sample α-diversity over time for each genotype. Boxplots show the spread of sample Simpsons α-diversity values for each of the four genotypes over the sampled time points. The values presented along the y-axis are the inverse α-diversity measure values (i.e., larger values [closer to one] represent samples with higher diversity and smaller values [closer to zero] represent samples with lower diversity). Related to Figure 4.

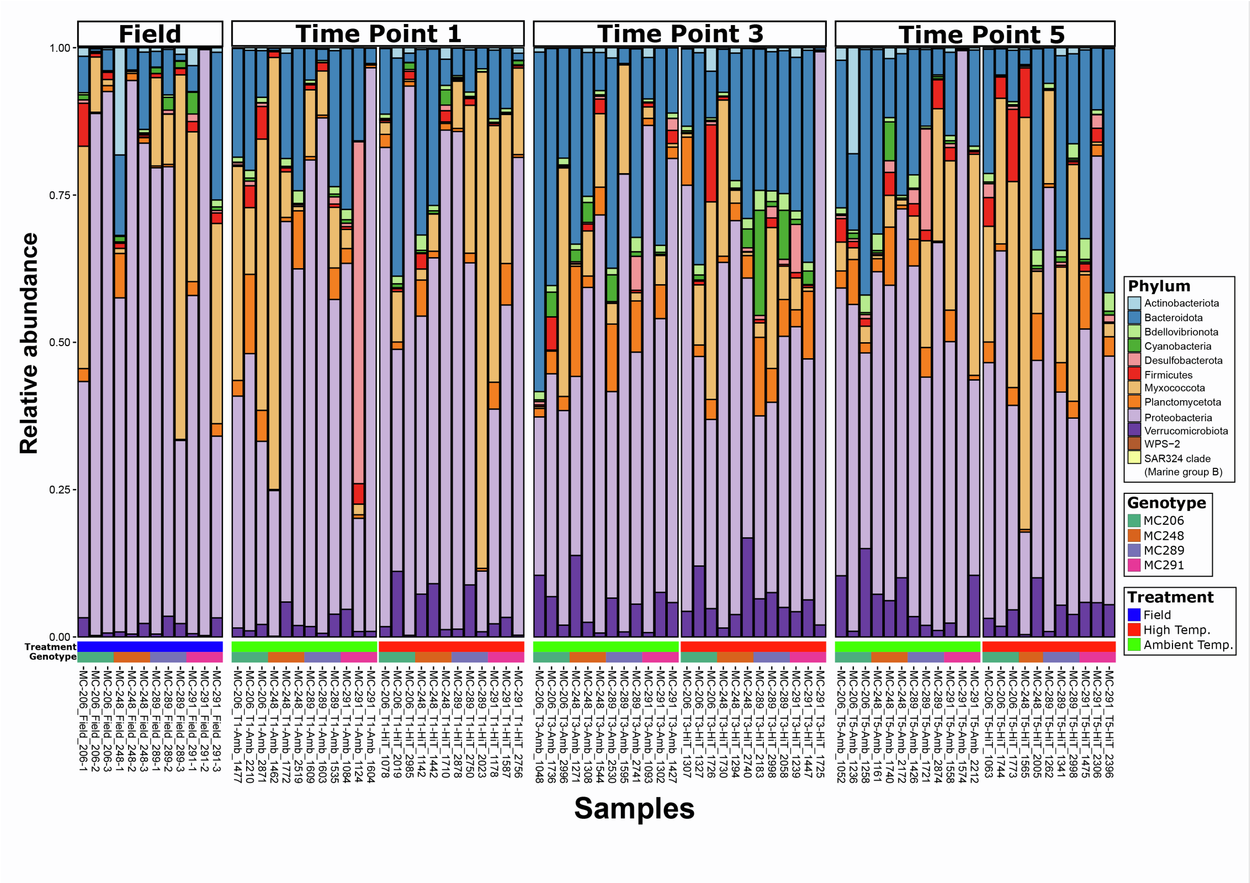

Figure S10. Top ten most abundant microbiome phyla in each sample across all four *M. capitata* genotypes. The treatment and genotype of each sample is shown as colored bars at the bottom of the figure. A legend describing the treatment, genotype, and phylum colors is shown on the right of the figure. Related to Figure 4.
